# Uncovering gene-family founder events during major evolutionary transitions in animals, plants and fungi using GenEra

**DOI:** 10.1186/s13059-023-02895-z

**Published:** 2023-03-24

**Authors:** Josué Barrera-Redondo, Jaruwatana Sodai Lotharukpong, Hajk-Georg Drost, Susana M. Coelho

**Affiliations:** 1 Department of Algal Development and Evolution, Max Planck Institute for Biology, Max-Planck-Ring 5, 72076 Tübingen, Germany; 2 Computational Biology Group, Department of Molecular Biology, Max Planck Institute for Biology, Max-Planck-Ring 5, 72076 Tübingen, Germany

**Keywords:** Gene age, Genomic phylostratigraphy, Homology detection failure, Evolutionary novelty, Taxonomically restricted genes, Gene emergence, Tree of life, Terrestrialization, Multicellularity

## Abstract

We present GenEra (https://github.com/josuebarrera/GenEra), a DIAMOND-fueled gene-family founder inference framework that addresses previously raised limitations and biases in genomic phylostratigraphy, such as homology detection failure. GenEra also reduces computational time from several months to a few days for any genome of interest. We analyze the emergence of taxonomically restricted gene families during major evolutionary transitions in plants, animals, and fungi. Our results indicate that the impact of homology detection failure on inferred patterns of gene emergence is lineage-dependent, suggesting that plants are more prone to evolve novelty through the emergence of new genes compared to animals and fungi.

**Supplementary Information:**

The online version contains supplementary material available at 10.1186/s13059-023-02895-z.

## Background

Most protein-coding genes of extant organisms descend from a limited set of founder genes already present in the last universal common ancestor (LUCA) of all living systems [1, 2]. Thus, evolutionary novelty at the molecular scale is largely driven by the duplication and neofunctionalization of preexisting genetic information [3]. Nonetheless, genomic studies over the past three decades show a pervasive number of genes with limited or untraceable gene homology [4–6], commonly known as “orphan” or taxonomically restricted genes (TRGs). The presence of TRGs is usually attributed to gene-family founder events, that is, the emergence of the last common ancestor of an extant family of protein-coding genes [7]. Several studies suggest that TRGs are associated with the emergence of novel morphologies [8, 9], immune defense mechanisms [10], and ecological specialization [11] across the tree of life. Proposed mechanisms that explain the birth of new gene families include neofunctionalization processes that modify the founder gene beyond recognition [4], the differential combination and fusion of protein folds and domains that predate the LUCA [12], or de novo gene birth from non-coding DNA [6]. However, the extent to which TRGs can be attributed to gene-family founder events has been extensively debated, since the lack of traceability of a gene can also explain the lack of detectable TRGs outside the evolutionary lineage under study [13–15]. With the advent of the Earth BioGenome Project, the scientific community is reaching a stage where representative genomes will be available for a major portion of eukaryotic lineages [16]. While presented as an unparalleled opportunity to study the evolutionary processes of genes and genomes across diverse evolutionary lineages [17], we lack a software solution that achieves high-confidence predictions of TRG origination events at a tree-of-life scale.

Genomic phylostratigraphy was initially introduced as a method to annotate gene founder events along the tree of life, often represented by taxonomic ranks [7]. Inferring the relative ages of genes helps to address evolutionary questions, such as the possible relationship between the emergence of TRGs and lineage-specific evolutionary novelties during major radiation events [18], how ontogenetic transcriptional patterns evolve [19, 20], whether new genes evolve faster than old genes [21], or at what rate the emergence of completely novel proteins is driven by de novo gene birth events [6]. While conceptually powerful, several studies have questioned the detection sensitivity of the phylostratigraphic approach [13–15, 22]. Gene ages may appear younger than they actually are due to gene prediction errors in the target database [23]. Previous approaches have overlooked contamination or horizontal gene transfer across lineages that can overestimate a gene’s age in a given organism [24]. Furthermore, previous implementations did not consider gene ages in terms of gene families, but assumed that dating individual genes extrapolates to the entire gene family [9, 25]. As such, the overall number of gene founder events is prone to be conflated by the subsequent duplication of a founder gene. Additionally, the computational burden of genomic phylostratigraphy limits its scalability. The pairwise sequence aligner BLASTP [26] is a gold standard tool to search gene homologs against sequence databases that is typically used for phylostratigraphic analyses [27, 28]. Phylostratigraphic analyses run with BLASTP have reported similar gene ages compared to slower but more sensitive profile-based methods, such as HMMER [29] or PSI-BLAST [28, 30]. Nevertheless, while faster than several alternative tools, a BLASTP search of a full set of organismal genes (approx. 5000 to 40,000 genes) against currently available public sequence databases can take up to several weeks or even months [17]. However, the biggest caveat of genomic phylostratigraphy is that small and fast-evolving genes are often wrongly annotated as young genes due to homology detection failure (HDF), i.e., the inability of pairwise local aligners to trace back distantly related homologs only due to neutral sequence divergence which results in spurious patterns of TRG birth [13, 15]. These important issues undermine the power of the original phylostratigraphic method, motivating several authors to propose key methodological improvements to accurately estimate gene-family founder events [9, 15, 23, 24, 28, 31, 32].

**Fig. 1:**
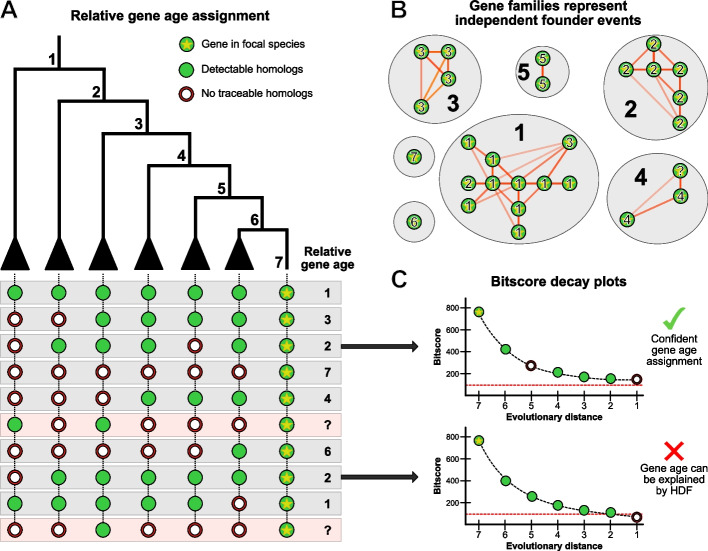
Overview of the methodological improvements to robustly estimate gene-family founder events. **A** Relative gene ages are inferred based on the principles of genomic phylostratigraphy, where each gene of the focal species is compared against a sequence database to find the most distantly related homolog. However, gene age inferences should also take into account the presence or absence of homologs throughout all the intermediate nodes between the focal species and the most distant homolog to distinguish putative gene losses from putative genome contaminations and horizontal gene transfer events. **B** Gene age inferences based on homology alone are expected to reflect the same founder event for other related genes. Thus, the age inferences of all loci in a gene family should not be regarded as independent values, but as a single evolutionary event. This compensates for the limited traceability of some paralogs within a gene family, whose ages are corrected as the oldest reliable age assignment in the family. **C** The estimated bitscore decay of genes as a function of evolutionary distance can be used to predict the expected bitscore of homologs in distantly related taxa where the gene has not been found. This prediction enables the calculation of homology detection failure (HDF) probabilities, which acts as a test to determine if a gene's absense beyond its most distantly related homolog can be attributed to HDF (the expected bitscore falls below the detectability threshold) or a gene-family founder event

Here, we present a conceptually redesigned gene-family founder inference method that employs the superior computational speed of our protein aligner DIAMOND v2 [17]. This method draws from the principles of genomic phylostratigraphy [7] to accurately infer gene ages, but extends its initial scope to account for gene-family founder events through the detection of gene families [33] and to account for HDF through the estimation of HDF probabilities [15] (Fig. 1). We use our methodology to revisit the putative pattern of TRG emergence associated with important evolutionary events in plants and animals, such as the transition to multicellularity in animals or terrestrialization in plants [4]. We also explore whether analogous TRG patterns are present in fungi. We calculate and investigate the gene age maps of 30 genomes across vastly different lineages within these three different eukaryotic kingdoms to test whether accounting for HDF changes the observed patterns of TRG emergence [15]. Finally, we evaluate the presence of ancient protein domains within these TRGs to estimate the relative contribution of gene duplication and domain reshuffling in TRG emergence compared to de novo gene birth.

## Results

### Addressing previous limitations of genomic phylostratigraphy with GenEra

**Fig. 2:**
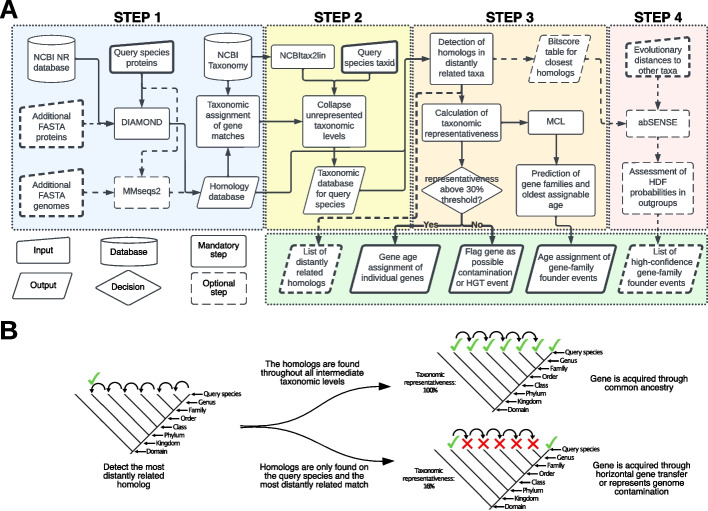
Gene-family founder detection framework implemented in GenEra. Overview of the pipeline for sensitive gene-family founder detection across the tree of life. **A** Flowchart of the command-line tool GenEra. Solid arrows/elements represent the mandatory steps in the pipeline, while the dashed arrows/elements represent optional steps to enrich the results. **B** Graphic representation of the rationale behind the taxonomic representativeness score. GenEra first performs a taxonomic trace-back to determine the most distantly related homolog to a query species, and then tracks back the presence of homologs in all the intermediate taxonomic levels, which helps to detect putative contaminants in the query proteome, horizontal gene transfer events between increasingly distantly related taxa, or false positive matches to the database

We address all major limitations and scalability of previous phylostratigraphic approaches while expanding its functionality by implementing a DIAMOND-fueled method to detect gene-family founder events (Fig. 2). Our pipeline can be used on the full set of genes from any species whose taxonomy is included in the NCBI database [34]. We provide this pipeline as an open-source command line tool called GenEra (https://github.com/josuebarrera/GenEra).

**Fig. 3:**
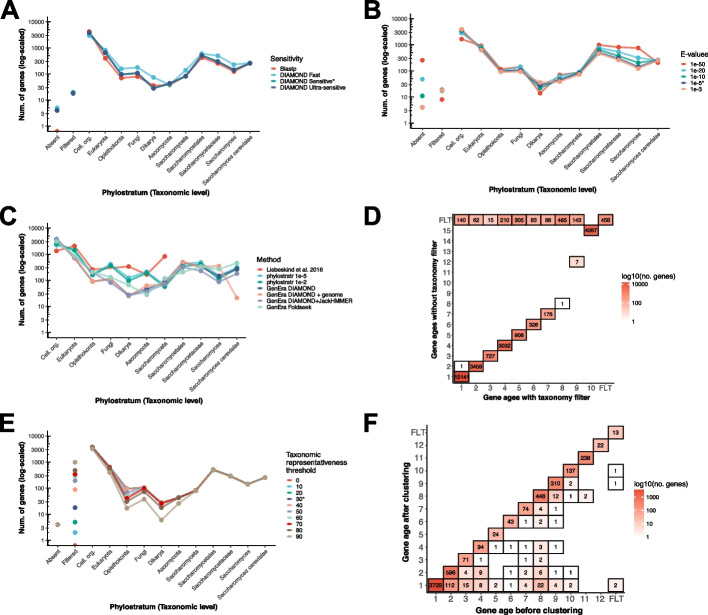
Benchmarking of GenEra through the analysis of *Saccharomyces* *cerevisiae* (**A–C** and **E, F**) and* Apostichopus japonicus *(**D**).** A** DIAMOND in ultra-sensitive and sensitive mode (*default parameter) generates a similar pattern of gene age assignment as the gold standard BLASTP while using the same e-value threshold of 10^−5^. The search sensitivity level does not influence the number of genes that are filtered through the taxonomic representativeness threshold (filtered) and has a negligible effect on the number of genes that fail to match themselves through pairwise alignment (absent). **B** The patterns of gene age assignment remain largely unaffected between a permissive e-value threshold of 10^−3^ and a more stringent threshold of 10^−5^ (*default parameter). Using more stringent thresholds (10^−10^ or lower) leads to an overrepresentation of TRGs at younger taxonomic levels. Lower e-value thresholds also increase the number of genes whose self-alignment cannot be detected (absent), thereby increasing the amount of false negative matches in the database. **C** GenEra can uncover deeper evolutionary relationships compared with previously published methods [24, 35], as seen in the number of genes that are traced back to the LUCA (cellular organisms). Using GenEra with additional 6-frame genome searches reduces the number of TRGs in the youngest taxonomic levels, from the species level up to the genus level, but older taxonomic levels are unaffected when including protein-against-genome data. Using JackHMMER increases the sensitivity at detecting homologs within older taxonomic levels, but shows little effect at finding homologs in the youngest taxonomic levels. Foldseek also increases the sensitivity at older levels but overestimates the number of genes at the species and genus levels. **D** Gene age assignments of *Apostichopus japonicus* before and after accounting for taxonomic levels lacking complete genomic data. The incomplete sampling of genomes across different taxonomic levels hinders gene age assignments, such as artificial patterns of gene absence that are erroneously filtered as contamination or HGT events (FLT). We established a parameter to exclude the taxonomic levels lacking genomic data, which improves the assignment of gene ages. **E** Taxonomic representativeness thresholds have a direct impact on the number of genes that can be assigned to a specific age (filtered). We established a default threshold of *30%, as lower values are bound to represent artifacts due to genome contamination and false positive matches while more stringent thresholds fail to account for gene losses and incomplete genome databases. **F** The clustering step helps to track down the founder events of some genes with limited traceability that share a common founder event with other paralogs of the same gene family, which is reflected in older gene age assignments

The first step of GenEra replaces BLASTP with the ultra-fast protein aligner DIAMOND v2 which we recently introduced for ultra-sensitive gene similarity assessments at a tree-of-life scale [17]. By default, BLAST and other sequence search algorithms limit the maximum number of top sequence hits that are reported in the analysis to the 500 best hits, which is an often-overlooked limitation that hinders the extent by which genes can be traced back to distantly related taxa. With exponentially growing sequence databases covering hundreds of thousands of species, 500 top hits can at best cover only 500 different subject species, thereby losing a significant proportion of age-assignable information. Using DIAMOND in sensitive mode instead of BLAST allows us to build a customized list of pairwise alignments against the entire NCBI non-redundant (NR) protein database, which harbors tens of thousands of genomes, alongside other user-defined protein datasets with an unlimited amount of sequence hits, generating results up to 8000 times faster than BLASTP-based approaches while reaching the same level of accuracy (Fig. 3A) [17]. We established an e-value threshold below 10^−5^ for a sequence hit to be considered a reliable true positive. The choice of this threshold was based on an extensive threshold-robustness study to test the influence of a diverse range of e-values on gene age assignments with the ultimate aim to determine the most robust e-value threshold when running GenEra in default mode. Indeed, a less stringent threshold does not improve the age assignment of genes and may lead to an increased rate of false positive age assignments, given the size of the NR database, while more stringent thresholds lead to an overestimation of TRGs (Fig. 3B).

 The constant overestimation of TRGs due to gene untraceability represents a valid concern when inferring gene ages [13, 15]. The standard gene age inferences that are performed using GenEra with unlimited sequence hits against the NR are able to trace back more distantly related homologs compared to other published methods that rely on a consensus approach [35] or pipelines that are restricted to a small set of genome comparisons [24] (Fig. 3C). Another issue that hinders gene age inferences is that spurious genome annotations and comparisons between annotations with different levels of quality and accuracy can overestimate the proportion of TRGs in the analysis [23, 31]. To address this shortcoming, GenEra includes an additional protein-against-genome search using Mmseqs2 [36] with its most sensitive parameters (s = 7.5) to reconfirm gene age assignments with an annotation-free approach solely based on six-frame alignments. We evaluated the impact of the six-frame search by adding alignments against 8 representative genome assemblies from each taxonomic level in *Saccharomyces cerevisiae*, adding to a total of 80 genomes (Additional file 1: Table S1). The age assignments of the youngest genes are pushed to older taxonomic levels when performing six-frame alignments, indicating that young gene age assignments are overestimated when not taking annotation errors into account [23]. However, older gene age assignments remain largely unaffected by annotation errors, demonstrating that protein-vs-genome searches are mostly impactful in assigning the age of the youngest TRGs (Fig. 3C). The sensitivity of pairwise sequence aligners has also been debated when inferring gene ages [25] so we incorporated an additional step with JackHMMER [29], through the Bio3D package in R [37], to reassess the gene ages that were predicted using DIAMOND. This additional step improved the detection of distantly related homologs across most taxonomic levels, but was less effective on the youngest taxonomic levels, as shown by instances of taxonomically inconsistent sequence hits against the database (Fig. 3C; Additional file 2: Fig. S1). Furthermore, the superposition of three-dimensional protein structures has become a viable alternative to both pairwise sequence aligners and HMM-based methods [38] ever since the advent of the AlphaFold protein structure database [39] and the development of scalable protein structure aligners [40]. Therefore, we integrated the fast structure aligner Foldseek [40] as an alternative to DIAMOND to identify protein homologs against the AlphaFold DB. Similar to JackHMMER, Foldseek can detect distantly related proteins in the oldest taxonomic levels, although it overestimates the number of young TRGs (Fig. 3C).

The second step of GenEra employs NCBItax2lin (available via https://github.com/zyxue/ncbitax2lin) to generate a lineage database that is used to associate the NCBI Taxonomy ID in the list of DIAMOND pairwise alignments with their hierarchical taxonomic identity in the NCBI Taxonomy database. The NCBI Taxonomy is a curated database that reflects the current knowledge of the relationships between all known organisms [34]. Hence, each taxonomic level in the lineage database often corresponds to a monophyletic group in a species tree, with the exception of certain taxonomic groups such as the paraphyletic suborder Microchiroptera [41] or the contested subkingdom Eumetazoa [42], which can be dealt with on a case-by-case basis. Thus, the NCBI Taxonomy allows GenEra to determine the evolutionary relationship between the matching genes from the sequence database and the query species. The lineage database that is generated by NCBItax2lin is not arranged in a hierarchical order, given that the taxonomic ranks are usually asymmetrical between different lineages in the NCBI Taxonomy database [43]. Thus, GenEra retrieves the correct taxonomic order from the NCBI server to rearrange the lineage database in a hierarchical order, following the taxonomic levels that are reported in the NCBI for the query species. 

Given the historical scopes and interests of the scientific community during the era of high-throughput sequencing, current genomic databases are still biased toward certain groups of organisms (e.g., crops and pathogens), while having only partial gene sets for others [44]. This complicates the detection of gene-family founder events, since having genomic data is required to reliably and systematically assign genes to a certain age. The absence of genomic data in certain taxonomic levels can be erroneously interpreted as systematic gene loss events that can lead to inconsistent phylogenetic patterns that are artificially introduced by database limitations. Particularly, this applies to lineages with limited genomic data. For example, as of January 20th of 2023, the class Holothuroidea (sea cucumbers) has 15 sequenced genomes uploaded to the NCBI database, where only one (*Apostichopus japonicus*) was uploaded with gene annotations [45]. Nonetheless, 5555 proteins in the NCBI belonging to Holothuroidea do not correspond to *Apostichopus japonicus*. These are mainly mitochondrial proteins (5213 sequences), but also include nuclear proteins (312 proteins) that span Holothuroidea and four other nested taxonomic groups leading to *Apostichopus japonicus*. Retaining those taxonomic levels severely impacts the gene age estimations of *Apostichopus japonicus*, showing artificial patterns of gene loss for most of the genes in these taxonomic groups (Fig. 3D). To address this issue, GenEra searches the entirety of sequence matches that were retrieved with DIAMOND and only retains the taxonomic levels for which at least one representative species matches more than 10% of the proteins in the query species for further analyses. This threshold was empirically established to exclude the organisms in the NR that are represented by only a few genes and not by genomic data (Additional file 2: Fig. S2). Using this threshold improves the detection of taxonomically inconsistent patterns of gene presence/absence by collapsing the taxonomic levels that would otherwise increase the proportion of artificial gene loss events in the analysis (Fig. 3D).

The third step of GenEra performs a taxonomic trace-back to determine the most distantly related lineage that matches each gene of the query species (Fig. 2B). Once the most distant homolog for a query protein is found, the pipeline calculates a taxonomic representativeness score to estimate the reliability of assigning a gene age based on this sequence match. The rationale for this procedure is to address another limitation of the original genomic phylostratigraphy, where the most distant hit was not reconfirmed at higher taxonomic levels but rather assumed, which created a systematic bias when dealing with contamination and horizontal gene transfer events. We now reconfirm hits at higher levels using our taxonomic representativeness metric (*L*) which is calculated as the presence of homologs in at least one representative species for each of the intermediate taxonomic levels between the most distantly related lineage and the query species (Fig. 2B). This metric assumes a ladder-like phylogenetic topology between the query species and the database species at each taxonomic level, a condition that is always met as long as the taxonomic levels that classify the query species represent monophyletic groups (Additional file 2: Fig. S3). The number of internode taxonomic levels with representative gene homologs (*RP*) is divided by the total number of taxonomic steps that separate the most distantly related match from the gene of the query species (*AP*) while excluding the youngest taxonomic level (usually the species level), since the presence of the gene in the query species already confirms its representativeness at that level:

$$L = 100 \times ( RP / (AP -1))$$

This gives a taxonomic representativeness score *L* with a scale from 100 to 100 × (1/(*AP* − 1)), which helps to flag genes that are only present in the query species and other distantly related taxa (Fig. 2B). Genes with low taxonomic representativeness are discordant with the concept of synapomorphy [46], where a homologous character (in this case, a gene) should be inherited to all the taxa that share a common ancestor. However, secondary losses of inherited genes should be expected to happen throughout the tree of life. Thus, the taxonomic representativeness score can be influenced by gene loss events in the genomes that act as representatives in the intermediate taxonomic levels, or due to the availability of only scarce and low-quality genomic data at certain taxonomic levels. To address this issue, we established a relaxed taxonomic representativeness threshold of 30%, so that only genes with a particularly low score are flagged as putative horizontal gene transfer events, contaminant sequences in the assembly that do not belong to the query species, or false positive matches against the database (Fig. 3E). Low levels of taxonomic representativeness are expected for cases of cross-kingdom and cross-domain contamination that are pervasive in genomic databases [47]. This score is reported for every gene in the query species, and the user can also establish a custom threshold that is appropriate for the dataset and taxon of interest.

GenEra can optionally report the best sequence hit (as defined by its bitscore) that can be assigned to the oldest taxonomic level for each query gene. This feature helps users to identify erroneous gene age assignments due to false positive matches, and to manually evaluate genes with low taxonomic representativeness. This feature also helps to identify candidate non-coding sequences from which potential de novo TRGs could have emerged when implementing a 6-frame genome search.

Once all the genes in a query species have been assigned to a certain age, GenEra performs an all-vs-all DIAMOND search of the query proteins against themselves to detect paralogs within the genome of the query species. The e-values of the all-vs-all DIAMOND search are transformed through a negative log10 transformation and are subsequently used for a clustering analysis to predict gene families using MCL [33]. GenEra uses the oldest assignable gene age for each of these gene clusters to estimate the number of gene-family founder events throughout the evolutionary history of the query species. This clustering step can assign genes to older ages, in accordance with the predicted founder event of their gene family (Fig. 3F).

GenEra has a fourth additional step to assess whether the gene age assignment of the query genes can be explained by HDF. Bitscores obtained through pairwise sequence alignments have been shown to decay exponentially as a function of evolutionary distance [15]. Given enough data points, the expected bitscore can be calculated for a given gene in a distantly related species when such gene is not detected, and thus compute the probability of not finding this gene as a consequence of bitscore decay alone [15]. When GenEra is given a list of pairwise evolutionary distances (e.g., substitutions per site in a phylogenetic tree) between the query species and other taxa in the database, it searches for the closest homolog in these species, which are defined as the highest bitscore matches to each of the query genes. GenEra uses the bitscore of these genes to calculate HDF probabilities using abSENSE [15] for all the species that lack any traceable homolog to each query gene in the target species. GenEra can use these probabilities to test the null hypothesis of untraceable homology for each gene that is assigned to a given taxonomic level. The ability of GenEra to test HDF for each taxonomic level is dependent on the taxonomic sampling that is given by the user, which is determined by the taxonomic sampling of the phylogeny that is used to calculate the evolutionary distances. Hence, the use of phylogenies at different taxonomic levels can be used by GenEra to test for HDF in gene-family founder events at different evolutionary scales. Once a gene is assigned to a certain age, GenEra analyzes the HDF probability of the closest species (as defined by their evolutionary distance to the query species) that belongs to the next taxonomic level, and labels the gene age assignment as a high-confidence gene-family founder event whenever the HDF probabilities fall below 0.05 in the outgroup (Additional file 2: Fig. S4). Gene-family founder events are considered high confidence if at least one of the genes in the family has HDF probabilities < 0.05 and the age of this gene is also the oldest assignable age for the family. Thus, GenEra can make an informed decision on whether the gene age assignments can be explained by gene-family founder events or through sequence divergence alone which makes these genes untraceable given their size and substitution rate [27].

### Major evolutionary transitions are associated with gene-family founder bursts

By improving genomic phylostratigraphy with a gene family clustering strategy and HDF probabilities, we were able to estimate the number of putative gene-family founder events throughout the plant, animal, and fungal lineages (Additional files 3, 4 and 5: Supplemental data 1–3). We analyzed 10 genomes for each of these lineages (Additional file 1: Table S2) to evaluate the common patterns of putative gene-family founder events that have been previously described using genomic phylostratigraphy with single genomes [4]. Then, we tested whether these putative gene-family founder events could be explained by HDF by calculating HDF probabilities in the closest outgroup for each taxonomic level for which we had evolutionary distances (see “Methods”).

**Fig. 4:**
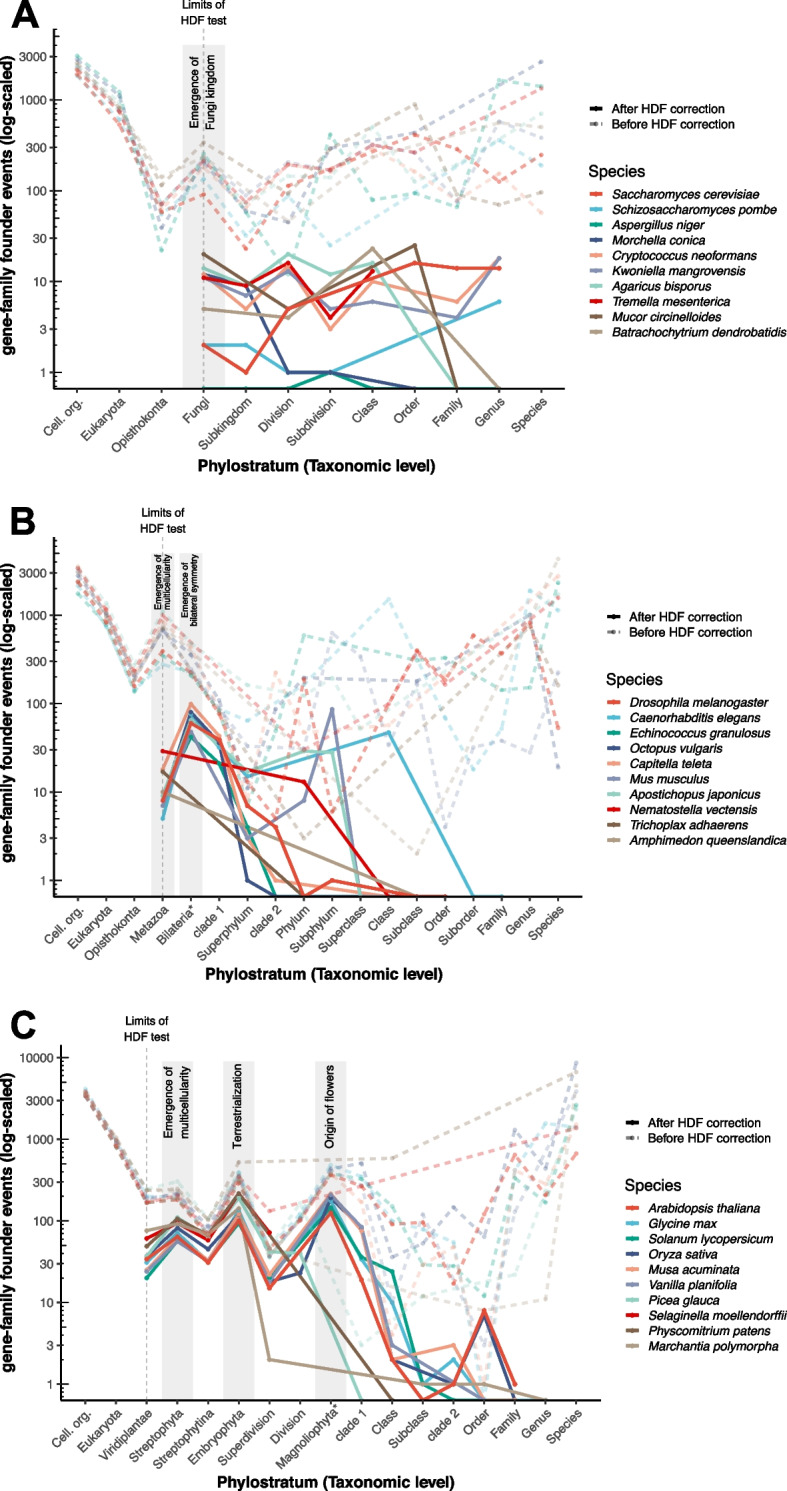
Detection of gene-family founder events at major evolutionary transitions in fungi, animals and plants. Overlapping plots of gene-family founder events before and after accounting for HDF (dashed lines and solid lines, respectively). The taxonomic hierarchies that are shared between all the species are named in the horizontal axis, while the taxonomic levels that differ between species are just labeled as their corresponding taxonomic ranks (see Additional file 6: Supplemental data 4). The limits of the HDF test for each kingdom, in accordance with the taxonomic sampling of the phylogenies that were used to extract evolutionary distances (see “Methods”), are marked with a vertical dashed line. **A** Gene-family founder events in fungi. The taxonomic level leading to the emergence of fungi exhibits a burst of gene-family founder events before the HDF test, but all the common patterns are lost after accounting for HDF. **B** Gene-family founder events in Metazoa. The taxonomic level leading to the emergence of Metazoa also shows a burst of gene-family founder events before the HDF test. The Metazoa burst fades after accounting for HDF, but the taxonomic level of Bilateria exhibits a burst after the HDF test for all bilaterian animals (Bilateria; *excluding *N. vectensis*, *T. adhaerens*, and *A. queenslandica*). **C** Gene-family founder events in Embryophyta. Plant genomes display a consistent pattern of gene-family founder events before and after accounting for HDF, with gene-family founder bursts associated with the emergence of multicellularity (Streptophyta), the conquest of land by plants (Embryophyta), and the origin of flowering plants (Magnoliophyta; *excluding *P. glauca*, *S. moellendorffii*, *P. patens*, and *M. polymorpha*)

Before calculating HDF probabilities, we found a consistent overrepresentation of putative gene-family founder events at the taxonomic levels that correspond to the crown node of land plants, animals, and fungi (Fig. 4). These gene age peaks were observed across vastly different taxonomic lineages within the same kingdom, revealing a common evolutionary signal. We found no evidence of whether this convergent pattern was correlated with the number of available genomes in the database at those taxonomic levels, as these levels can have a vastly different number of representative genomes depending on the species that is analyzed (Additional file 1: Table S3).

However, these patterns of gene-family founder events change after filtering the dataset by HDF probabilities. The total number of putative gene-family founder events diminished between one and two orders of magnitude in all the analyzed species after retaining the high-confidence gene ages that could not be explained by HDF. Fungi lost any discernible pattern of gene-family founder events that could be traced back to a particular evolutionary transition after accounting for HDF, including the putative TRG overrepresentation at the kingdom level (Fig. 4A). Likewise, the signal associated with the emergence of Metazoa is lost in the high-confidence gene-family founders, but the transition to bilateral symmetry (Bilateria) is consistently enriched in high-confidence gene-family founder events on all the bilateral animals in our dataset (Fig. 4B). We analyzed the biological activity of these TRGs by looking at the gene annotation of *Drosophila melanogaster*. We detected the emergence of the Ninjurin A-C genes, the Disconnected gene, the Dampened gene, and the gene family composed of the Gurken, Keren, and Spitz genes.

The patterns of gene-family founder events in plants remained consistent despite predicting a smaller number of gene-family founder events. The most consistent bursts of gene-family founder events in plants were found in Streptophyta when green algae transitioned to complex multicellularity [48], in embryophytes when plants conquered the land [49], and in angiosperms, when plants evolved flowers [50] (Fig. 4C). We inspected the gene annotations of *Arabidopsis thaliana* to evaluate the biological activity of these TRGs.

Some of the successful gene-family founder events that were identified as high-confidence Streptophyta TRGs include a family of Basic Helix-Loop-Helix (bHLH) transcription factors [51], the COBRA-like gene family that acts as key regulators of cell-wall expansion in the meristems [52], a family of auxin canalization proteins that regulate plant growth through auxin transport [53], and the BRASSINAZOLE-RESISTANT family of transcription factors that modulate brassinosteroid signaling in plants [54]. Surprisingly, the ARABIDILLO and ULTRAPETALA gene families were found as Streptophyta TRGs, with putative homologs in the charophyte algae *Klebsormidium nitens* (GAQ84482.1 and GAQ90507.1, respectively).

The high-confidence gene-family founder events that were linked to the emergence of embryophytes include a family of F-box/kelch-repeat proteins that regulate the biosynthesis of phenylpropanoids [55], the group 2 of late embryogenesis abundant (LEA) proteins that are involved in plant response to osmotic and oxidative stress due to desiccation [56], two groups of bHLH transcription factors [51], a gene family that contains MORPHOGENESIS OF ROOT HAIR 6 (MRH6), a gene family that contains Piriformospora indica-insensitive protein 2 (PII-2), the SOSEKI gene family that regulates cell polarity in early plant development [57], and the LONGIFOLIA gene family that is involved in leaf development [58].

Within the gene-family founder events in angiosperms, we found class III of Ovate family proteins (OFP) and the family of paclobutrazol resistance (*PRE*) genes. However, most of the TRGs in this taxonomic level belong to genes that are uncharacterized in *A. thaliana*. The founder event of the MADS-box gene family could be traced back to the LUCA.

## Discussion

Gene founder events facilitate evolutionary innovations [8–11]. Determining the timing of these events is therefore paramount for evolutionary research. Such inference is not trivial, since previous attempts to estimate TRG birth have overlooked the effects of HDF and other biases [4, 7–9, 18]. While these initial efforts were useful for investigating general processes of evolution, such as the assessment of transcriptome age during development [19, 20], they lack the detection sensitivity to decouple founder events of entire gene families from patterns of gene untraceability. For this reason, we developed GenEra to provide the community with a sensitive and computationally optimized approach for gene-family founder detection across the tree of life. To demonstrate the versatility of GenEra, we analyzed 30 genomes from plants, animals, and fungi to capture the broad diversity of gene-family founder events in these lineages. We show that GenEra can be used on any potential eukaryotic genome and provide extensive documentation to facilitate its swift adoption in the life science community.

Our benchmarking procedures show that HMM-based methods can retrieve more distantly related homologs across most gene age categories compared to using only DIAMOND. However, despite its increased sensitivity, we did not find a strong impact of HMM-based methods on the overall patterns of gene age assignment, supporting the conclusions of previous studies [28]. Moreover, we found an increased number of taxonomically inconsistent matches on the genes assigned to the youngest taxonomic levels. Unsupervised HMM-based methods are prone to model corruption that can increase the number of false positive matches when searching for homologs [59], which in turn might explain these taxonomically inconsistent patterns. While increased alignment sensitivity helps to trace back more distantly related homologs, it does not solve the underlying problem of homology detection failure during gene age assignments [15, 28] that should be explicitly tested when estimating gene ages. Structural alignments do show important differences in gene age assignment compared to sequence alignments, but they cannot be readily interpreted as being superior in terms of gene age inference. As with HMM-based methods, we found an increased sensitivity on genes that were previously assigned to old taxonomic ranks, but we also found an overestimation of young TRGs compared to both pairwise sequence alignments and HMM-based alignments. Structural alignments lose sensitivity when dealing with highly disordered proteins [60], a characteristic that is known to be conflated with young TRG assignments [13, 25, 61]. Thus, a combination of pairwise sequence alignments coupled with a 6-frame genome search seems to be the most effective strategy to analyze young and disordered genes, while HMM-based methods and structural alignments may be more adequate when studying old and highly structured genes. All these options are implemented on GenEra to suit the needs of each research project.

The origin of TRGs has sparked important debates over the last decade regarding the processes of gene birth [4–6, 13–15, 22, 27]. A high proportion of gene age assignments in our dataset could be explained by HDF, as previously reported [13–15]. It is important to acknowledge that gene age assignments that fail the HDF test should not be interpreted as not belonging to their estimated taxonomic level, but rather that we cannot reject the null hypothesis of untraceable homology in more distantly related lineages [15]. This is particularly true for short and fast-evolving genes that are prone to fail the HDF test [15] but are also expected to have arisen recently, given that previously validated de novo genes are usually shorter and have fewer exons compared to old genes [11, 62, 63]. Finding conserved motifs and domains outside the boundaries of TRGs is a conceivably compelling evidence to discard de novo birth scenarios. The vast majority of the high-confidence TRGs we detected contain highly conserved protein domains and motifs that are consistently found throughout the tree of life. Such is the case for the bHLH motif that is found in transcription factors across all eukaryotes [64], the DIX domain in SOSEKI genes that are also conserved throughout eukaryotes [57], the ARMADILLO repeat domain in ARABIDILLO genes that can be found in animals [65], or transmembrane domains found throughout all cellular organisms [66]. These TRGs cannot be explained by HDF [13, 15], nor through de novo gene birth, as previously suggested [5]. Our observations support the idea of gene duplication [3] and of protein modularity, where gene-family founder events result from the differential fusion of preexisting folds and domains [12], whose tertiary structure acquired the property to fold during the postulated era of the RNA and peptide world [1, 2]. These domain-containing TRGs were coincidentally found as multi-copy gene families, suggesting that evolutionary old protein folds and domains were optimized through natural selection to perform their biological activity [1, 2], ensuring the evolutionary success of these TRGs. Despite the minor role of de novo gene birth in TRG emergence, the study and validation of successful de novo founder events should be of particular interest for evolutionary research, as these events can help us uncover the processes that shape evolutionary novelty at the molecular level [67].

Our results before the HDF test retrieved analogous peaks of gene age assignments in plants and animals that have been previously described by Tautz and Domazet-Lošo [4] and could extend their insights by detecting a kingdom-level peak in fungi. The consistency of these peaks throughout several species with vastly different evolutionary histories and biological traits (e.g., free-living organisms and parasites, unicellular and multicellular fungi, plants with haploid-dominant and diploid-dominant life cycles, bilateral-symmetric and non-bilateral-symmetric animals) points toward a biological basis of such a convergent pattern. However, the biological interpretation of TRG patterns should always be considered cautiously. These TRG peaks have been previously interpreted as bursts of genomic novelty that have accompanied some important diversification events throughout the evolutionary history of these lineages [4, 9], but we found that the overrepresentation of TRGs at the emergence of animals and fungi disappears after accounting for HDF, suggesting that these peaks may be driven by untraceable homology beyond those taxonomic levels [15], rather than gene-family founder events or any other source of molecular novelty.

The emergence of animals and fungi is associated with their independent emergence of multicellularity [68] and the diversification bursts that followed this key evolutionary innovation [69]. Diversification events have long been known to correlate with molecular substitution rate accelerations [70–72], even though the exact causal relationship between both phenomena remains underexplored [73]. If substitution rates are correlated with diversification events, we would expect a large proportion of the genes in the genome to become untraceable beyond these major diversification bursts. Accordingly, our analyses show a pattern of gene untraceability that is linked to the emergence and the diversification bursts of these two eukaryotic kingdoms. Therefore, we propose that these gene age assignment peaks are driven by substitution rate accelerations that were linked to the diversification bursts that accompanied these major evolutionary transitions in animals and fungi. Although gene emergence likely influenced these evolutionary transitions in the tree of life, our results indicate that gene-family founder events may not be as pervasive in the emergence of evolutionary novelties such as multicellularity in opisthokonts compared to the co-option of ancient gene families that already existed in the LUCA, such as transcription factors, cell-adhesion proteins, and cell-signaling genes, which likely drove biological novelty through novel regulatory pathways [74, 75]. Furthermore, recent studies suggest multiple origins of multicellularity in fungi through vastly different evolutionary processes compared to animals or plants [75]. This likely blurs any common pattern between molecular innovations and the transition to multicellularity in fungi. A more in-depth analysis of fungal genomes might elucidate key gene-family founder events in this eukaryotic lineage and may resolve downstream incongruencies such as whether transcriptomic hourglass patterns mark fruit body development across fungal species [76, 77].

We found a consistent overrepresentation of gene-family founder events in Bilateria. The emergence of Bilateria is defined by a change in developmental patterns that resulted in the evolution of bilateral symmetry. Among our reported gene-family founder events, we found Gurken, Spitz, and Dampened as Bilateria TRGs. These genes are all involved in the establishment of the anterior–posterior and dorsal–ventral polarities and neurogenesis during development [78–80]. Likewise, the protein Disconnected is involved in the formation of the nervous system and the connection of the visual nerve to the brain [81].

Our results show that three major evolutionary transitions in plants are associated with the evolution of entire new gene families. The observed pattern of TRG birth in plants is conserved even after accounting for HDF, suggesting that plants are indeed prone to evolve novel traits through the emergence of new genes. The frequency of gene-family founder events in plants could be driven by the propensity of their genomes to undergo structural rearrangements and whole-genome duplications [82]. This could be the case for the origin of flowering plants, which was accompanied by a whole-genome duplication event [83]. Our results are consistent with an orthogonal approach by Bowles et al., who report an independent burst of gene novelty in the phylogenetic branches leading to Streptophyta and Embryophyta [8], even though that study did not account for HDF, which likely inflated the number of predicted TRGs at those taxonomic levels. Streptophytes, which include land plants and charophytes, have been proposed to share a common emergence of complex multicellularity [8, 48]. Complex multicellularity has been linked with the expansion of transcription factors, the emergence of an internal communication system between cells [68] and, in the case of plants, the emergence and expansion of cell-wall remodeling proteins [48]. Coincidentally, our analysis detected gene-family founder events in bHLH transcription factors [51], BRASSINAZOLE-RESISTANT transcription factors [54], COBRA-like genes [52], and auxin canalization proteins [53]. Furthermore, the emergence of auxin canalization proteins and BRASSINAZOLE-RESISTANT genes likely contributed to the establishment of an internal communication system between cells in multicellular streptophytes through the regulation of the basic hormone-receptor systems that predate the evolution of multicellularity [84]. We found putative ARABIDILLO and ULTRAPETALA homologs among charophyte algae, even though these gene families were previously reported as embryophyte and angiosperm TRGs, respectively [65, 85]. ARABIDILLO genes have been co-opted to modulate different developmental processes in plants through abscisic acid signaling [65], while ULTRAPETALA genes interact with the trithorax group of angiosperms to coordinate flower development through chromatin-dependent transcriptional regulation [85]. If the homologs found in *Klebsormidium nitens* are reliable, this would suggest an early role of ULTRAPETALLA and ARABIDILLO homologs in streptophyte evolution [86].

The evolution of land plants (Embryophyta) is intertwined with an increased morphological complexity compared to other streptophytes. The emergence of SOSEKI genes probably conferred plants with cell-polarization mechanisms to ensure the correct development of complex multicellularity [57]. The LONGIFOLIA gene likely played an additional role in the emergence of complexity in land plants through the development of leaves [58]. The emergence of embryophytes has also been associated with the emergence of several defense mechanisms to cope with the abiotic stresses that characterize the transition from water to land, such as ultraviolet (UV) radiation, drought, and temperature fluctuations [49]. Accordingly, we found an F-box/kelch-repeat gene-family founder event in Embryophyta, whose gene members regulate phenylpropanoid biosynthesis [55]. The production of phenylpropanoids has long been recognized as a crucial adaptation in plants that allowed them to survive the effects of UV radiation on land [49]. The emergence of group 2 LEA proteins would have helped plants to cope with drought stress [56] as they transitioned from water to land. The role of rooting structures and their association with mycorrhizal fungi have also been proposed as important innovations in land plants [49]. We detected two bHLH groups in our analysis, which have been shown to coordinate the development of rhizoids and roots in plants [87]. We also detected MRH6 as an embryophyte TRG, which is involved in root hair development [88]. Furthermore, PII-2 is known to promote plant growth and seed production through its interaction with the mycorrhizal fungus *Piriformospora indica *[89], whose detection as an embryophyte TRG supports the role of plant-fungus interactions in the transition from water to land [49].

The emergence of flowers and fruits are major evolutionary innovations in angiosperms that changed the ecological dynamics of terrestrial life [50]. Many genes that regulate flower development are known to belong to evolutionary old gene families, such as the MADS-box genes [90]. Accordingly, our analysis retrieved the founder event of MADS-box genes in the LUCA. However, our results also detected the founder event of the class III OFP genes, which are also involved in the development of fruits [91]. Most of the founder events we detected in angiosperms belong to uncharacterized genes with unknown biological activity. The experimental study of these TRGs should allow further research to shed new light on the evolution of flowering plants. While we focused on early evolutionary transitions across three distinct eukaryotic kingdoms, we expect future studies to harness GenEra at different evolutionary scales, in underexplored lineages and for other biological questions such as the transcriptional conservation during development and the nature of genetic novelty.

## Conclusions

Our results show that decoupling confident gene age assignments from HDF can lead to a conservative estimation of gene-family founder events. Further advances in detecting gene-family founder events should focus on HDF correction, given that current methods with higher alignment sensitivity do not solve the issue of gene untraceability [28]. The putative patterns of gene emergence at key evolutionary transitions can be lost after accounting for HDF, as observed in opisthokonts, or it can be mostly congruent with the patterns that are retrieved from genomic phylostratigraphy, as in the case of plant genomes. We argue that the propensity of plant genomes to undergo and survive large genomic rearrangements provides them with higher genomic evolvability that is reflected in their patterns of gene-family founder events. The consistency of our results with previous studies on the emergence of these widely studied evolutionary transitions highlights the power of this approach to accurately detect molecular innovations through gene-family founder events. Turning our gaze to the rest of the tree of life, we anticipate that other major evolutionary transitions are also marked by distinct patterns of gene-family founder events, such as in other multicellular eukaryotes like the red and brown algae [92].

## Methods

The benchmarking analyses were performed using the genome of *S. cerevisiae* [93] and *A. japonicus* [45]. We compared the results of GenEra to those of phylostratra [24] and the consensus method of Liebeskind et al. [35] by extracting the gene age values that were published from their respective manuscripts. These analyses were performed with different annotation versions of *S. cerevisiae*, so we only compared the genes that were common between all the annotations.

We downloaded representative genomes of plants, animals, and fungi from the UniProt reference proteomes to study the patterns of gene-family founder events throughout these major eukaryotic lineages by using GenEra (Additional file 1: Table S2). We ran homology detection analyses for each gene of these species against 44,637 genomes that are publicly available in the NR as of 2 July 2022 (Additional file 1: Table S3). We chose 10 representative taxa across the taxonomic diversity of each of these three kingdoms to revisit the previously observed peaks in gene founder events associated with the diversification of animals and land plants [4] and to determine whether this same pattern arises in fungi. We collapsed the Eumetazoan taxonomic level (i.e., all animals excluding Porifera) from our animal analysis since recent evidence suggests that Eumetazoa is paraphyletic [42].

We extracted the evolutionary distances from previously reported phylogenies using the ape package in R [94] to calculate HDF probabilities at different taxonomic levels and evaluate the proportion of gene families that can be confidently assigned to gene-family founder events. For the kingdom Fungi, we used 81 evolutionary distances from a maximum likelihood tree [95] encompassing several evolutionary distances from our 10 target genomes (Additional file 1: Table S4), including *Fonticula alba* and other opisthokonts as outgroups to test gene-family founder events up until the Fungi level. For Metazoa, we used 43 evolutionary distances from a posterior consensus Bayesian tree [42] comprising a large portion of the animal phyla (Additional file 1: Table S5) and which includes *Monosiga brevicollis* and *Salpingoeca rosetta* as outgroups [96] to test gene-family founder events at different taxonomic levels up to Metazoa. For Embryophyta, we used 61 evolutionary distances (Additional file 1: Table S6) from a posterior consensus Bayesian tree [97] that incorporates several plant genomes, as well as green algae and red algae, which helped us test gene-family founder events up until the Viridiplantae level. All the gene families who had HDF probabilities < 0.05 in the closest outgroup were considered high-confidence TRGs that resulted from gene-family founder events.

## Supplementary Information

**Additional file 1:** **Table S1.** List of genome assemblies that were used to test the impact of a 6-frame protein-vs-genome search in the gene age estimations of *S. cerevisiae*. **Table S2.** List of genome assemblies spanning three major eukaryotic kingdoms, with representative genomes encompassing the biological diversity of these lineages. **Table S3.** Number of available species with genomes on the NCBI database that represent each taxonomic level for each species in the analysis. **Table S4. **Evolutionary distances that were used to calculate homology detection failure probabilities in fungi. **Table S5.** Evolutionary distances that were used to calculate homology detection failure probabilities in animals. **Table S6.** Evolutionary distances that were used to calculate homology detection failure probabilities in land plants.

**Additional file 2: Fig. S1.** Comparison of gene ages predicted with DIAMOND and JackHMMER in the proteome of *S. cerevisiae*. **Fig. S2.** Distribution of taxa in the NR with sequence matches against the proteome of *S. cerevisiae*. **Fig. S3.** Example of a ladder-like topology when dealing with monophyletic groups. **Fig. S4.** Decoupling gene founder events from homology detection failure (HDF).

**Additional file 3. **Raw results for the 10 analyzed fungal species using GenEra. The sheets within the file are named and ordered after the species taxonomy ID. The file contains the gene ages and the gene-family founder events, both before and after accounting for homology detection failure, for each of the 10 species.

**Additional file 4.** Raw results for the 10 analyzed animal species using GenEra. The sheets within the file are named and ordered after the species taxonomy ID. The file contains the gene ages and the gene-family founder events, both before and after accounting for homology detection failure, for each of the 10 species. **Note:** The animal-specific gene-family founder events that are described and discussed in the manuscript can be found on the sheet “3702_HDF_founder_events”.

**Additional file 5.** Raw results for the 10 analyzed plant species using GenEra. The sheets within the file are named and ordered after the species taxonomy ID. The file contains the gene ages and the gene-family founder events, both before and after accounting for homology detection failure, for each of the 10 species. **Note:** The plant-specific gene-family founder events that are described and discussed in the manuscript can be found on the sheet “7227_HDF_founder_events”.

**Additional file 6.** Input tables that were used to generate the plots from Fig. 4, including the information of which taxonomic levels were collapsed for each species and which taxonomic levels were compared across species for each kingdom.

**Additional file 7.** Review history.

## Data Availability

**Implementation** GenEra is available on GitHub [98] and Zenodo [99]. The source code is released under the GNU General Public License v3.0. The GitHub repository contains all the details on how to install and run GenEra on Linux operating systems through the command line. **Datasets** The accession numbers of the genome assemblies that were used to benchmark the 6-frame GenEra analysis can be found in Additional file 1: Table S1. The accession numbers of the 30 analyzed eukaryote species can be found in Additional file 1: Table S2. Our results are available in Additional files 3, 4, 5 and 6: Supplemental data 1–4.
