# Supplementary material for: Uncovering gene-family founder events during major evolutionary transitions in animals, plants and fungi using GenEra
Source: Genome Biol. 2023 Mar 24;24:54. doi: 10.1186/s13059-023-02895-z (PMC10037820; doi:10.1186/s13059-023-02895-z)
Supplement: Supplementary file 2 — Additional file 2: Fig. S1. Comparison of gene ages predicted with DIAMOND and JackHMMER in the proteome of S. cerevisiae. Fig. S2. Distribution of taxa in the NR with sequence matches against the proteome of S. cerevisiae. Fig. S3. Example of a ladder-like topology when dealing with monophyletic groups. Fig. S4. Decoupling gene founder events from homology detection failure (HDF). [file 13059_2023_2895_MOESM2_ESM.docx]

**Supplementary figures**


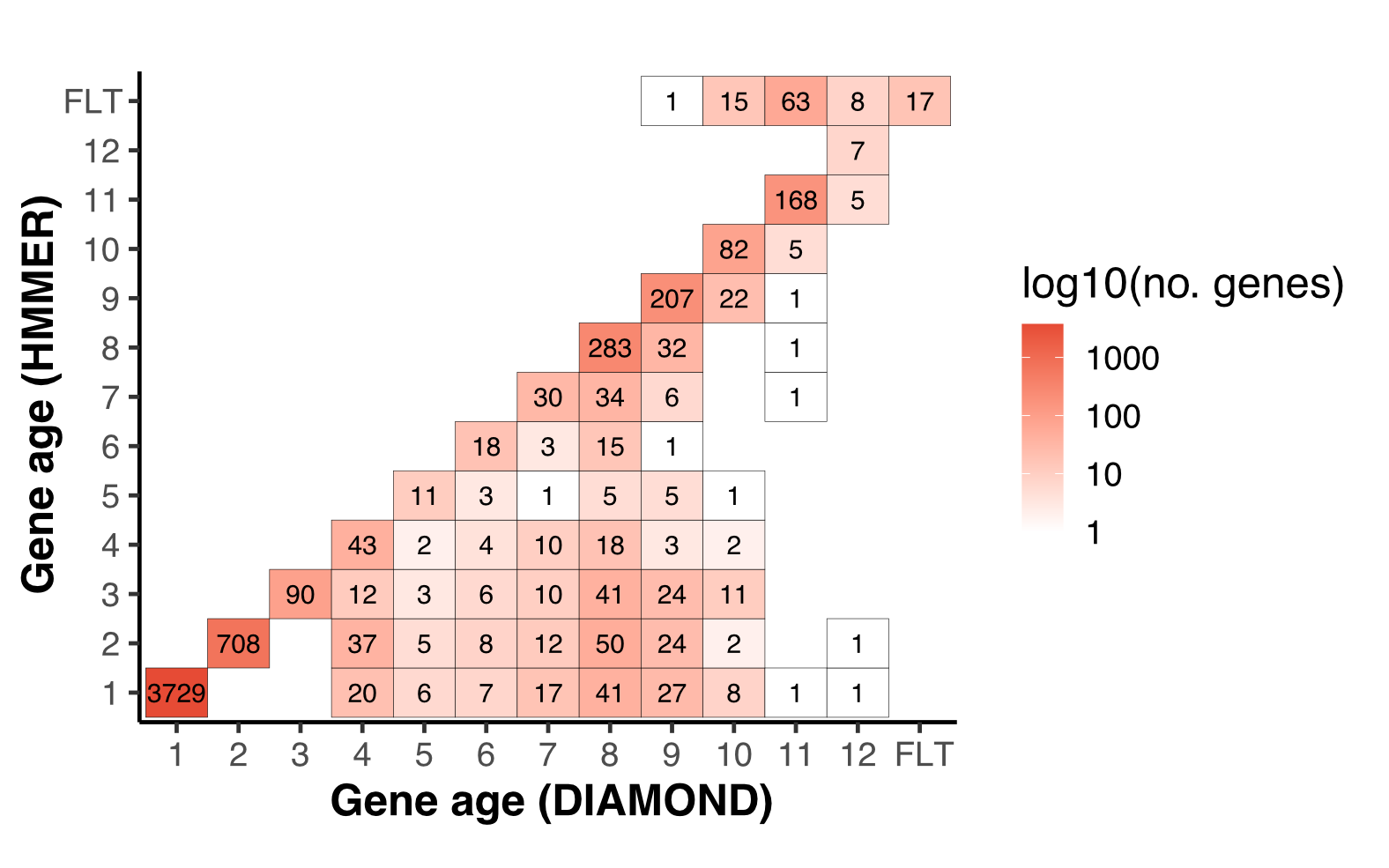
**Figure S1. Comparison of gene ages predicted with DIAMOND and JackHMMER in the proteome of *S. cerevisiae*.** The inferred gene ages using DIAMOND [ 1 ] were reassessed by running JackHMMER [ 2 ] from the fourth oldest taxonomic level onwards. JackHMMER is able to detect more distant homologs on most of the intermediate taxonomic levels, but its efficacy is reduced on the youngest taxonomic levels. Furthermore, many of the reassessed genes in the youngest taxonomic levels were filtered (FLT) due to the detection of taxonomically-inconsistent sequence hits against the database (taxonomic representativeness scores < 30).


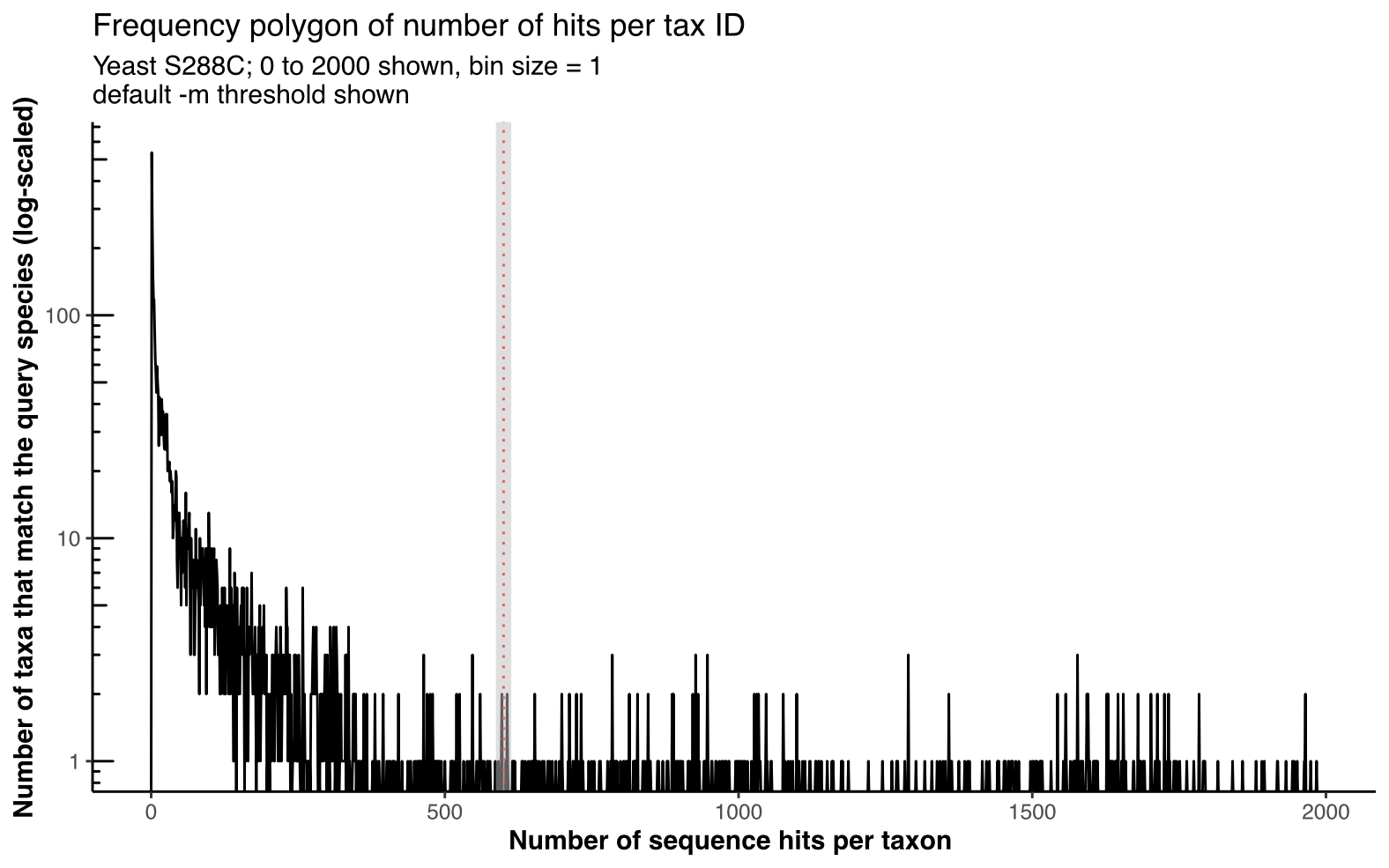


**Figure S2. Distribution of taxa in the NR with sequence matches against the proteome of *S. cerevisiae*.** Most of the taxa in the NCBI non-redundant database contain 50 or less proteins (*e.g.*, sequence data generated for phylogenetic studies), which could lead to an unreliable gene age assignment. An empirical threshold of 10% total hits against the query proteome can successfully detect these cases (red dashed line). This allows GenEra to assign ages only to the taxonomic levels where genomic data is available.


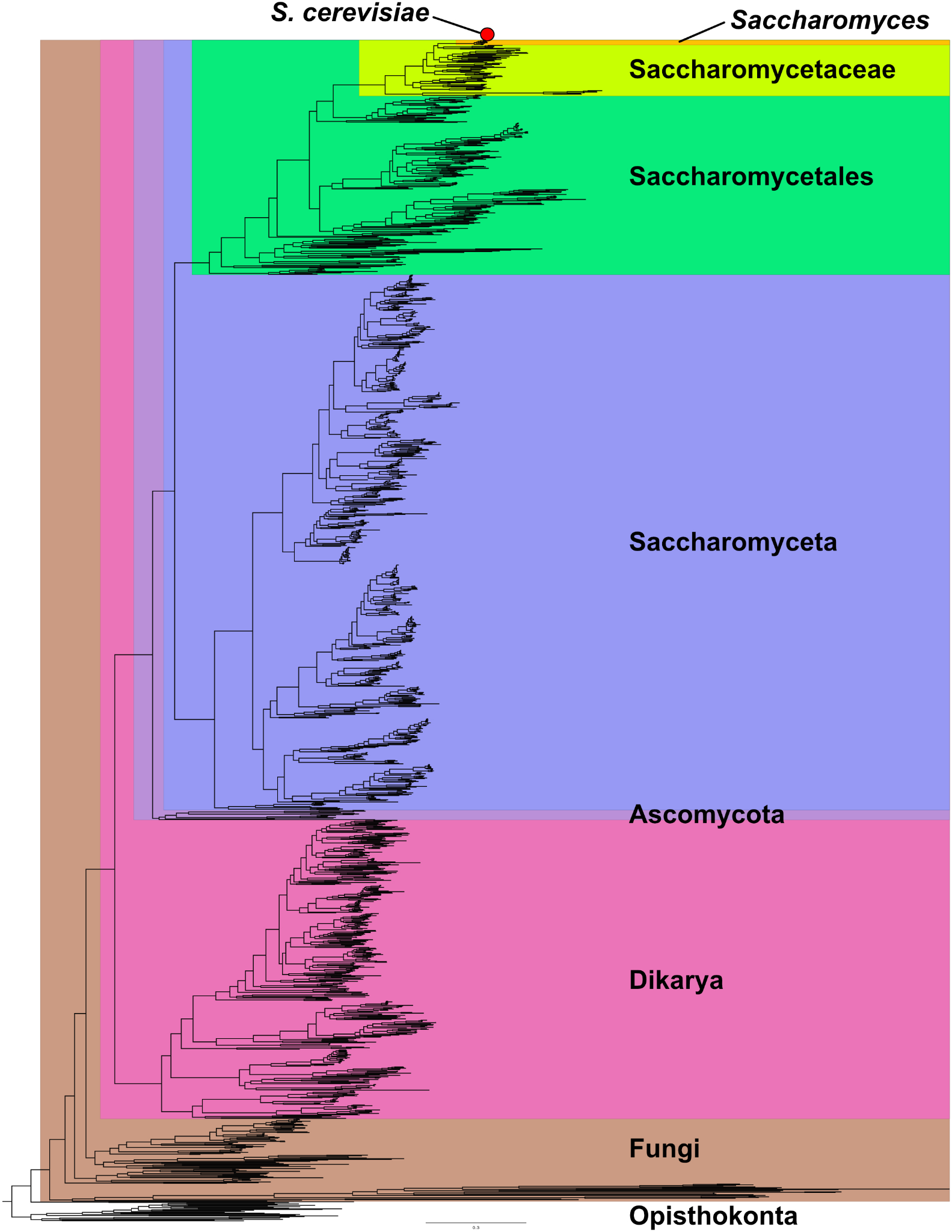


**Figure S3. Example of a ladder-like topology when dealing with monophyletic groups.** We use the phylogeny of Fungi published by Li *et al*. [ 3 ] to depict the way GenEra interprets evolutionary relationships between species, with *S. cerevisiae* as the query species. As long as the taxonomic ranks represent monophyletic groups, the evolutionary relationships between species can be interpreted as a ladder-like topology, where all the inner nodes within each taxonomic rank are treated as polytomies. Therefore, only the nodes that define the taxonomic groups become relevant for the assessment of gene ages and the calculation of taxonomic representativeness scores.

**
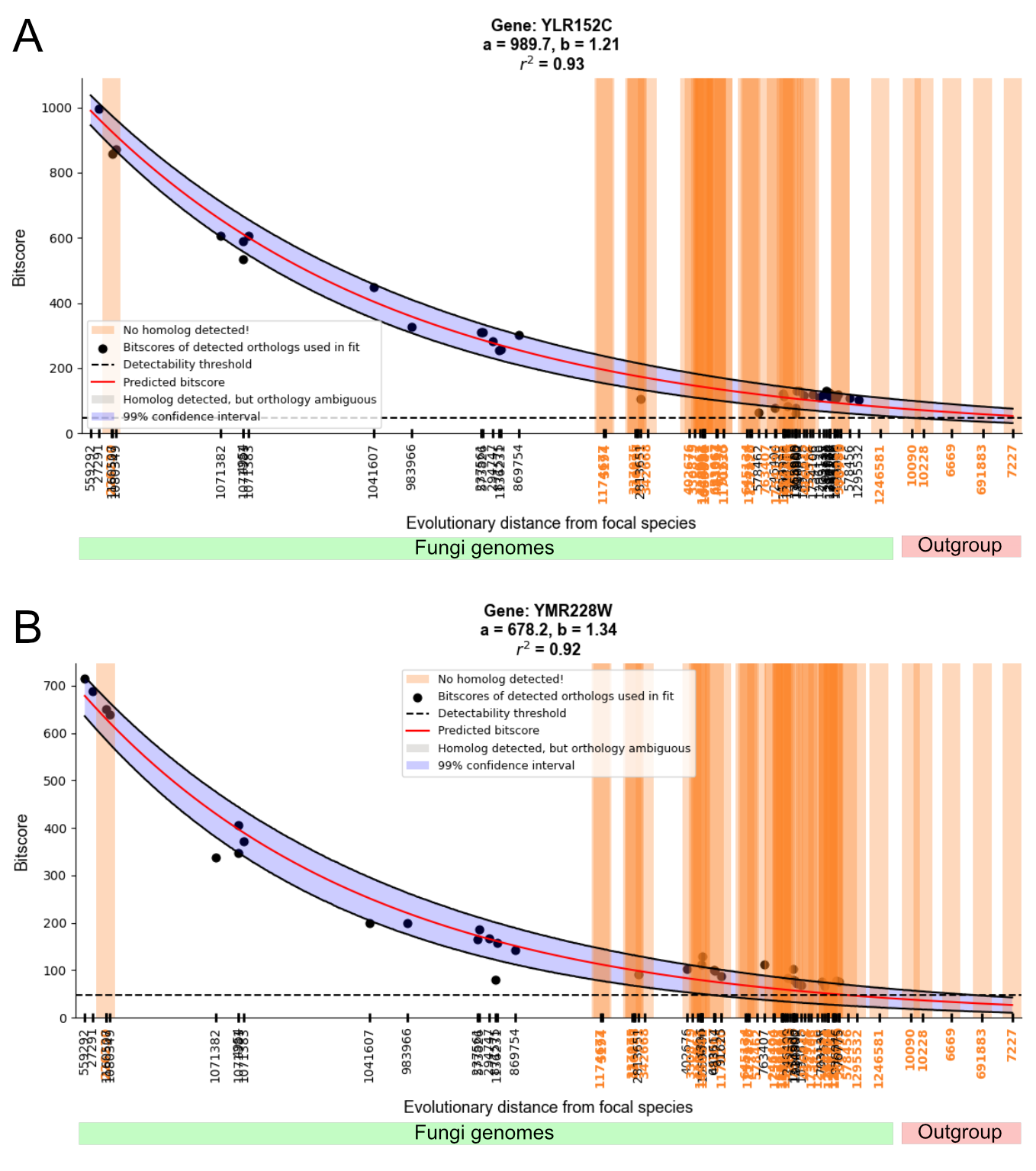
**

**Figure S4. Decoupling gene founder events from homology detection failure (HDF).** Bitscore decay plots as a function of evolutionary distance in two genes of *S. cerevisiae* across different fungal species (green bar) and other closely related opisthokonts (red bar). Both genes lack detectable homologs outside of the Fungi kingdom. Each species in the horizontal axis is represented by its NCBI Taxonomy ID. The bitscore values were retrieved from a DIAMOND search against the NR using GenEra, while the pairwise evolutionary distances (substitutions per site) were retrieved from a previously published maximum likelihood tree [ 3 ]. The bitscore prediction and the detection failure probabilities were calculated using abSENSE [ 4 ]. **A** Bitscore decay of the gene YLR152C, which shows homolog genes only within the Fungi kingdom, but is predicted to have detectable bitscore values in *Fonticula alba* and in other closely-related animal genomes (detection failure probability of 0.01 in the outgroup species). The absence of YLR152C homologs outside of Fungi is thereby regarded as a gene founder event. **B** The gene YMR228W appears to be restricted to the Fungi kingdom, but its expected bitscore has decayed to below the detectability threshold (indicated by the dashed line) not only in Fonticula alba but also in other closely-related animal genomes (detection failure probability of 1 in the outgroup species). Therefore, the seeming absence of this gene outside the Fungi kingdom can be explained by HDF.

**References**

1. Buchfink B, Reuter K, Drost H-G. Sensitive protein alignments at tree-of-life scale using DIAMOND. Nat Methods. 2021;18(4):366–8.
2. Johnson LS, Eddy SR, Portugaly E. Hidden Markov model speed heuristic and iterative HMM search procedure. BMC Bioinformatics. 2010;11:431.
3. Li Y, Steenwyk JL, Chang Y, Wang Y, James TY, Stajich JE, et al. A genome-scale phylogeny of the kingdom Fungi. Curr Biol. 2021;31(8):1653-1665.e5.
4. Weisman CM, Murray AW, Eddy SR. Many, but not all, lineage-specific genes can be explained by homology detection failure. PLoS Biol. 2020;18(11):e3000862.
