# Supplementary material for: Uncovering gene-family founder events during major evolutionary transitions in animals, plants and fungi using GenEra
Source: Genome Biol. 2023 Mar 24;24:54. doi: 10.1186/s13059-023-02895-z (PMC10037820; doi:10.1186/s13059-023-02895-z)
Supplement: Supplementary file 7 — Additional file 7. Review history. [file 13059_2023_2895_MOESM7_ESM.docx]

Review History

**First round of review**

**Reviewer 1**

**Were you able to assess all statistics in the manuscript, including the appropriateness of statistical tests used?** Yes, and I have assessed the statistics in my report.

**Comments to author:**

This manuscript presents a new method, GenEra, to date the emergence of gene family founder events at increased accuracy. The approach uses the much faster and sensitive Diamond aligner to retrieve all hits for a comprehensive taxonomic gene assignment. It searches both the set of annotated genes and the respective genomes to account for incomplete annotations. A taxonomy consistency score is computed that allows GenEra to discard genes that were likely horizontally transferred or are due to genome assembly contamination. Homology detection failure, a common issue with inferring gene emergency, is also explicitly taken into consideration. Finally, the age of a gene family is determined by the oldest age of any of its members, which is more robust than inferring individual gene age.

The manuscript is well written, the method is well explained and every step is carefully optimized. For example, only species that are represented by sequenced genomes are considered. The authors performed an analysis covering plants, animals and fungi, which revises previous conclusions about peaks of gene family emergence and suggests that evolutionary innovations are associated with increased substitution rates, which in turn limits traceability in earlier-branching clades.

I have only a few comments.

1) page 11: Taxonomy is not phylogeny. E.g. NCBI still distinguishes between mega- and microchiroptera, the latter being a paraphyletic group that is no longer supported by phylogenetic analyses. While there is no question that NCBI taxonomy is useful and sufficient for their analysis, I would suggest to avoid overly strong language and maybe write in line 228 that a taxonomic level *often* corresponds to a clade.

2) page 9: It is great to see that not only annotated genes present in NR are searched for, but that also a 6 frame genomic scan is conducted. To make it clear that entire genomes (and not just any nucleotide subset) is searched, I would suggest to replace 'nucleotide' by 'genome' in the phrases 'protein-to-nucleotide search' and '6 frame nucleotide search' (Fig 3 legend).

3) I believe one major aspect to increase accuracy is dating a gene family by considering the age of its members, instead of dating individual genes; however, there was no benchmark to show this effect. Maybe this can be added, e.g. by comparing the final age of a gene family to the spread of ages of its members and calculating what percentage of family members would underestimate the age?

4) The last results section shows that considering HDF can drastically change the results of peak gene family emergences. After taking HDF into account, the functional relevance of gene families (e.g. phenylpropanoids synthesis) that emerged during important evolutionary transitions is used as an argument that these genes contributed to these innovations. If that is the case, one would expect that gene families discarded by HDF (they are potentially older) should not have functions that would contribute to such innovations. In other words, one would expect that the two groups of gene families that were initially considered to have emerged in say land plants, and then failed (group 1) and survived (group 2) the HDF test, have different functional profiles and enrichments. Maybe this can be explored.

**Reviewer 2**

**Were you able to assess all statistics in the manuscript, including the appropriateness of statistical tests used?** Yes, and I have assessed the statistics in my report.

**Comments to author:**

Overall, this is a very nice paper. It takes a straightforward approach to producing a method to perform phylostratigraphy at large scale more quickly and with higher accuracy than has previously been possible. The major advances are 1. Improved speed due to use of DIAMOND instead of eg BLASTP; 2. A more principled and systematic method for throwing out instances of HGT/contamination that would lead genes to look older than they truly are; and 3. The integration of an existing method to control for homology detection failure, which would lead genes to look younger than they truly are. The method itself is useful, and the results from its application are important.

I have three concerns, which, although will likely be relatively minor for the authors to implement, to me seem non-negotiable points. The second and third may be based on my own misunderstanding; if so, I would appreciate clarification.

First, If the name of the game is to find homologous proteins - which, at the end of the day, is the game here - then I do not see any justification for not using a more sensitive homology search tool (eg profile-based methods like HMMER) than BLASTP/DIAMOND. I do understand that these profile-based tools are so much slower than DIAMOND that using them at scale is not practical. But it seems to me that there is nothing standing in the way of using the fast DIAMOND as a first filter, and then performing a HMMER search only on those proteins that are inferred by GenEra to be young (how young may be a user-set parameter, with some reasonable default, eg not found beyond the first 4 phylostrata). It is standard practice in phylostratigraphy for these methods not to be used, so this manuscript is not uniquely deficient in this respect -- but to my mind this has always been an enormous failing of the field, leading to many false positives.
The authors claim that eg HMMER has similar sensitivity to BLASTP, but this is obviously untrue, based on extensive benchmarking studies (see eg work by Finn/Eddy and the PFAM database) and large numbers of individual cases in which HMMER successfully finds homologs where BLASTP fails; moreover, the claim is based only on a single citation in which the object of study were not real sequences but a small set of sequences whose evolution was simulated in silico.
There is just no way around the fact that if your goal is to identify the most remote possible homologs, you ought to use the best possible tools for identifying homologs. I would therefore like to see a final HMMER/other search step added to the pipeline.

Second, I have concerns about the step described in lines 237-247, in which hits that appear in taxonomic levels containing no organisms that have hits to less than 10% of the query species' proteins are thrown out. It seems to me that this is appropriate if no hits are found in such taxonomic levels: the absence of homologs in those levels could well be explained by the lack of available sequence data, such that concluding that the gene is absent at that level is unfounded. But if hits are found in such taxonomic levels (eg in a taxon where all sequence data is partial, not resulting from whole genomes), I don't see a reason for excluding this taxonomic level: that the genomes are partial does not seem to invalidate the finding that there is a homolog there. (If I am not understanding this method correctly, I would appreciate clarification in the text.)

Third, I may be missing it, but the results described in the text - the assignments of gene age for the different focal species in the plants, animals, and fungi - do not seem to be available anywhere. Though the manuscript says that they are in Supplemental Information, I cannot find them anywhere (I only have Supp Tables 1-6, none of which contain this information). I also do not see them in the GitHub Repository. These must be available as a condition of publication. If I have missed them or some technical error has occurred in transmitting the files, please clarify/correct.

One more minor concern:
- I am not clear on the biological rationale behind the TR score, the authors' method of filtering out cases of HGT or sequence contamination. It seems to me that the relevant quantity should be something like the number of independent losses that would have had to take place for the gene to have been present in the common ancestor of the distant homolog-possessing taxon and the query species: if this number is high, i.e. a large fraction of all the taxa under consideration would have to have lost the gene, such losses are implausible, and the data point to HGT/contamination. It is not totally clear to me from the description that the TR score amounts to this. It instead counts the percent of monophylys in which the gene is present/absent. While these are the same in the simple, ladder-like topology of Figure 2B, it is not obvious to me that they will always be identical in real phylogenies. I may be wrong; if so, clarification in the text would be useful. If I am correct, can the authors clarify or say something about this choice?

**Authors Response**

**Point-by-point responses to the reviewers’ comments:**

Reviewer #1: This manuscript presents a new method, GenEra, to date the emergence of gene family founder events at increased accuracy. The approach uses the much faster and sensitive Diamond aligner to retrieve all hits for a comprehensive taxonomic gene assignment. It searches both the set of annotated genes and the respective genomes to account for incomplete annotations. A taxonomy consistency score is computed that allows GenEra to discard genes that were likely horizontally transferred or are due to genome assembly contamination. Homology detection failure, a common issue with inferring gene emergency, is also explicitly taken into consideration. Finally, the age of a gene family is determined by the oldest age of any of its members, which is more robust than inferring individual gene age.

The manuscript is well written, the method is well explained and every step is carefully optimized. For example, only species that are represented by sequenced genomes are considered. The authors performed an analysis covering plants, animals and fungi, which revises previous conclusions about peaks of gene family emergence and suggests that evolutionary innovations are associated with increased substitution rates, which in turn limits traceability in earlier-branching clades.

I have only a few comments.

1) page 11: Taxonomy is not phylogeny. E.g. NCBI still distinguishes between mega- and microchiroptera, the latter being a paraphyletic group that is no longer supported by phylogenetic analyses. While there is no question that NCBI taxonomy is useful and sufficient for their analysis, I would suggest to avoid overly strong language and maybe write in line 228 that a taxonomic level *often* corresponds to a clade.

RESPONSE 1: We appreciate the reviewer’s acknowledgement about the usefulness of the NCBI taxonomy for tree-of-life scale analyses. We agree that the presence of paraphyletic groups in the NCBI taxonomy separates this approach from a formal phylogenetic analysis, and we understand that paraphyletic groups can even be problematic for the detection of gene-family founder events. We have modified the manuscript as suggested by the reviewer, while including an additional disclaimer regarding paraphyletic and polyphyletic groups (lines 229-234):

*“Hence, each taxonomic level in the lineage database often corresponds to a monophyletic group in a species tree, with the exception of certain taxonomic groups such as the paraphyletic suborder Microchiroptera (42) or the contested subkingdom Eumetazoa (43), which can be dealt with on a case-by-case basis. Thus, the NCBI Taxonomy allows GenEra to determine the evolutionary relationship between the matching genes from the sequence database and the query species.”*

2) page 9: It is great to see that not only annotated genes present in NR are searched for, but that also a 6 frame genomic scan is conducted. To make it clear that entire genomes (and not just any nucleotide subset) is searched, I would suggest to replace 'nucleotide' by 'genome' in the phrases 'protein-to-nucleotide search' and '6 frame nucleotide search' (Fig 3 legend).

RESPONSE 2: We appreciate the reviewer’s suggestion, and made changes accordingly throughout the manuscript (lines 168, 177, 205, 207, 308 and 454).

3) I believe one major aspect to increase accuracy is dating a gene family by considering the age of its members, instead of dating individual genes; however, there was no benchmark to show this effect. Maybe this can be added, e.g. by comparing the final age of a gene family to the spread of ages of its members and calculating what percentage of family members would underestimate the age?

RESPONSE 3: We thank the reviewer for this great suggestion. We have now plotted the differences in gene ages before and after clustering (Fig. 3F; lines 219-221):

*“F) The clustering step helps to track down the founder events of some genes with limited traceability that share a common founder event with other paralogs of the same gene family, which is reflected in older gene age assignments.”*

We also describe the effects of the clustering step in the Results section of the manuscript (lines 314-317):

*“GenEra uses the oldest assignable gene age for each of these gene clusters to estimate the number of gene-family founder events throughout the evolutionary history of the query species. This clustering step can assign genes into older ages, in accordance with the predicted founder event of their gene family (Fig. 3F).*”

4) The last results section shows that considering HDF can drastically change the results of peak gene family emergences. After taking HDF into account, the functional relevance of gene families (e.g. phenylpropanoids synthesis) that emerged during important evolutionary transitions is used as an argument that these genes contributed to these innovations. If that is the case, one would expect that gene families discarded by HDF (they are potentially older) should not have functions that would contribute to such innovations. In other words, one would expect that the two groups of gene families that were initially considered to have emerged in say land plants, and then failed (group 1) and survived (group 2) the HDF test, have different functional profiles and enrichments. Maybe this can be explored.

RESPONSE 4: We appreciate the reviewer’s insight on this topic. Our original argument was indeed that most of the TRGs that pass the HDF test are mostly genes whose evolution has been studied in detail, and have been shown to be relevant for the evolutionary transitions with which they coincide. It was also a way of showing how some genes that have been previously attributed to the emergence of these lineages are supported even after testing for homology detection failure. We would in principle agree with the reviewer’s intuition that discarded genes should not show this pattern. There is, however, one caveat to consider: old genes can also be co-opted into new functions, and an old gene can always contribute to a specific synapomorphy that appears later on throughout evolution (e.g., Ryan & Baxevanis, 2007; Sebé-Pedrós et al., 2010; Moody et al., 2016; Flores-Sandoval et al., 2018). Therefore, we are not sure how to implement this idea in a testable and systematic manner. However, if the reviewer has a suggestion, we will be very glad to explore this matter.

Reviewer #2: Overall, this is a very nice paper. It takes a straightforward approach to producing a method to perform phylostratigraphy at large scale more quickly and with higher accuracy than has previously been possible. The major advances are 1. Improved speed due to use of DIAMOND instead of eg BLASTP; 2. A more principled and systematic method for throwing out instances of HGT/contamination that would lead genes to look older than they truly are; and 3. The integration of an existing method to control for homology detection failure, which would lead genes to look younger than they truly are. The method itself is useful, and the results from its application are important.

I have three concerns, which, although will likely be relatively minor for the authors to implement, to me seem non-negotiable points. The second and third may be based on my own misunderstanding; if so, I would appreciate clarification.

First, If the name of the game is to find homologous proteins - which, at the end of the day, is the game here - then I do not see any justification for not using a more sensitive homology search tool (eg profile-based methods like HMMER) than BLASTP/DIAMOND. I do understand that these profile-based tools are so much slower than DIAMOND that using them at scale is not practical. But it seems to me that there is nothing standing in the way of using the fast DIAMOND as a first filter, and then performing a HMMER search only on those proteins that are inferred by GenEra to be young (how young may be a user-set parameter, with some reasonable default, eg not found beyond the first 4 phylostrata). It is standard practice in phylostratigraphy for these methods not to be used, so this manuscript is not uniquely deficient in this respect -- but to my mind this has always been an enormous failing of the field, leading to many false positives.

The authors claim that eg HMMER has similar sensitivity to BLASTP, but this is obviously untrue, based on extensive benchmarking studies (see eg work by Finn/Eddy and the PFAM database) and large numbers of individual cases in which HMMER successfully finds homologs where BLASTP fails; moreover, the claim is based only on a single citation in which the object of study were not real sequences but a small set of sequences whose evolution was simulated in silico. 
There is just no way around the fact that if your goal is to identify the most remote possible homologs, you ought to use the best possible tools for identifying homologs. I would therefore like to see a final HMMER/other search step added to the pipeline.

RESPONSE 5: We acknowledge the validity of the reviewer’s concern regarding the limited sensitivity of pairwise local sequence aligners to find distantly related homologs, thereby influencing the detection of putative TRGs. Indeed, profile HMM methods (*e.g.*, PSI-BLAST or HMMER) have long been acknowledged to be more sensitive than pairwise local sequence aligners, as rightfully stated by the reviewer (Madera & Gough, 2002). Nonetheless, our argument was not that profile HMM methods are equally sensitive as DIAMOND in finding distantly-related homologs, but rather that gene age inferences are not drastically different when using profile HMM methods over pairwise local sequence aligners (Moyers & Zhang, 2018). We understand that this misunderstanding was conveyed by how we wrote the manuscript, so we modified this in the new version of the manuscript (lines 78-80):

“*Phylostratigraphic analyses run with BLASTP have reported similar gene ages compared to slower but more sensitive profile-based methods, such as HMMer* [*(29)*](https://sciwheel.com/work/citation?ids=1253554&pre=&suf=&sa=0) *or PSI-BLAST* [*(30)*](https://sciwheel.com/work/citation?ids=27750&pre=&suf=&sa=0)”

For example, one study using genomic phylostratigraphy with HMMER replicated the observed trend in hydrophobicity among gene ages that has been previously reported using BLASTp (James et al., 2021). Protein properties such as hydrophobicity, substitution rate or protein size are known to be conflated with gene age as a consequence of gene untraceability (Moyers & Zhang, 2015; Weisman *et al*., 2020), meaning that increased sensitivity does not solve the basic underlying problem of genomic phylostratigraphy, which is homology detection failure (Weisman *et al*., 2020).

We were initially hesitant to implement profile HMM methods due to HMMER being orders of magnitude slower than DIAMOND, severely limiting its scalability. Another problem of profile HMM methods are their propensity to model corruption when not being manually supervised (Pearson et al., 2017), which might lead to spurious results when implemented in an automated fashion. However, the reviewer’s suggestion of sampling a subset of genes in a genome makes this implementation feasible. We now include an option in GenEra to re-analyze DIAMOND’s gene age assignments using JackHMMER (Johnson *et al*., 2010) against the UniProt database. By default, this option will analyze from the second taxonomic level onwards, but due to time constrains we ran it from the 4^th^ taxonomic level onwards on *S. cerevisiae*, as suggested by the reviewer, to benchmark this addition against the results obtained with DIAMOND and with other methods that are based on genomic phylostratigraphy (Fig. 3C; Fig. S1, lines 920-927):

“*Figure S1. Comparison of gene ages predicted with DIAMOND and JackHMMER in the proteome of S. cerevisiae. The inferred gene ages using DIAMOND were reassessed by running JackHMMER from the fourth oldest taxonomic level onwards. JackHMMER is able to detect more distant homologs on most of the intermediate taxonomic levels, but its efficacy is reduced on the youngest taxonomic levels. Furthermore, many of the reassessed genes in the youngest taxonomic levels were filtered (FLT) due to the detection of taxonomically-inconsistent sequence hits against the database (taxonomic representativeness scores < 30).*”

An alternative to profile HMM methods are structural aligners, which are allegedly more sensitive compared to pairwise local sequence aligners, while avoiding the limitations of profile HMM methods. Additionally, structural alignments are able to detect homologs that are highly divergent in sequence similarity, sometimes outperforming sequence-based homology approaches like DIAMOND and HMMER (Julian *et al*., 2021). Therefore, we propose to also integrate Foldseek (van Kempen *et al*., 2022) to GenEra as an alternative to DIAMOND to perform protein structural alignments without compromising the scalability of our pipeline with tree-of-life datasets. We traced homologs in *S. cerevisiae* against the Alphafold Protein Structure Database (Varadi *et al.*, 2022), and we integrated this analysis as benchmarking in new Fig 3C.

It should be noted that GenEra can be fed with the results from any desired tool, as long as the output is given with all the relevant information in a tab-delimited table (query ID, target ID, e-value, bitscore and target taxonomy ID), so GenEra is flexible to the needs of the user.

We describe the incorporation of these methods and their benchmarking within the manuscript (lines 177-190, 204-210 and 435-457):

“*The sensitivity of pairwise sequence aligners has also been debated when inferring gene ages (25) incorporated an additional step with JackHMMER (29), through the Bio3D package in R (39), to reassess the gene ages that were predicted using DIAMOND. This additional step slightly increases the detection of distantly related homologs at most taxonomic levels, its efficacy is reduced on the youngest taxonomic, where we found instances of taxonomically inconsistent sequence hits against the database (Fig. 3C; Fig. S1). Furthermore, the superposition of three-dimensional protein structures has become a viable alternative to both pairwise sequence aligners and HMM-based methods (40) ever since the advent of the AlphaFold protein structure database (41) and the development of scalable protein structure aligners (33). Therefore, we integrated the fast structure aligner Foldseek (33) as an alternative to DIAMOND to identify protein homologs against the AlphaFold DB. Similar to JackHMMER, Foldseek can detect distantly-related proteins in the oldest taxonomic levels, although it overestimates the amount of young TRGs (Fig. 3C).*”

“*Using GenEra with additional 6-frame genome searches reduces the number of TRGs in the youngest taxonomic levels, from the species-level up to the genus-level, but older taxonomic levels are unaffected when including protein-against-genome data. Using JackHMMER increases the sensitivity at detecting homologs within older taxonomic levels, but shows little effect at finding homologs in the youngest taxonomic levels. Foldseek also increases the sensitivity at older levels, but overestimates the number of genes at the species and genus level.*”

“*Our benchmarking procedures show that HMM-based methods can retrieve more distantly related homologs across most gene age categories compared to using only DIAMOND. However, despite its increased sensitivity, we did not find a strong impact of HMM-based methods on the overall patterns of gene age assignment, as previously suggested in simulation studies (28). Moreover, we found an increased number of taxonomically-inconsistent matches on the genes assigned to the youngest taxonomic levels. Unsupervised HMM-based methods are prone to model corruption that can increase the number of false positive matches when searching for homologs (59), which in turn might explain these taxonomically inconsistent patterns. While increased alignment sensitivity helps to trace back more distantly related homologs, it does not solve the underlying problem of homology detection failure during gene age assignments (15,28) that should be explicitly tested when estimating gene ages. Structural alignments do show important differences in gene age assignment compared to sequence alignments, but they cannot be readily interpreted as being superior in terms of gene age inference. As with HMM-based methods, we found an increased sensitivity on genes that were previously assigned to old taxonomic ranks, but we also found an overestimation of young TRGs compared to both pairwise sequence alignments and HMM-based alignments. Structural alignments lose sensitivity when dealing with highly disordered proteins (60), a characteristic that is known to be conflated with young TRG assignments (13,25,61). Thus, a combination of pairwise sequence alignments coupled with a 6-frame genome search seems to be the most effective strategy to analyze young and disordered genes, while HMM-based methods and structural alignments may be more adequate when studying old and highly structured genes. All these options are implemented on GenEra to suit the needs of each research project.*”

Finally, we uploaded to Github a new version of GenEra (v1.1.0), with the incorporation of Foldseek and JackHMMER as alternatives to our default DIAMOND alignment (https://github.com/josuebarrera/GenEra).

Second, I have concerns about the step described in lines 237-247, in which hits that appear in taxonomic levels containing no organisms that have hits to less than 10% of the query species' proteins are thrown out. It seems to me that this is appropriate if no hits are found in such taxonomic levels: the absence of homologs in those levels could well be explained by the lack of available sequence data, such that concluding that the gene is absent at that level is unfounded. But if hits are found in such taxonomic levels (eg in a taxon where all sequence data is partial, not resulting from whole genomes), I don't see a reason for excluding this taxonomic level: that the genomes are partial does not seem to invalidate the finding that there is a homolog there. (If I am not understanding this method correctly, I would appreciate clarification in the text.)

RESPONSE 6: We understand the reviewer’s concerns, given the way we conveyed this part of the pipeline in the manuscript. As the reviewer mentions, the taxonomic levels with < 10% of hits to the query proteome are “thrown out”, or “collapsed” in phylogenetic jargon. It is true that, even if sequence hits are found on only 1% of the query genes, they represent evidence of the presence of those genes on those taxonomic levels. However, these cases usually represent either highly incomplete genomes within that taxonomic level on the database, or the presence of a few genes in the database that are usually used for phylogenetic analyses from species that lack any other sequences on the database aside from those selected markers. This fact seems innocuous on itself, until one considers the effect of near-empty taxonomic levels on the assessment of taxonomic representativeness (TR) scores. Since these taxonomic levels lack complete genomic data, almost all the genes in the analysis would be artificially “absent” in those taxonomic levels and would be affected by having lower TR scores, which would result in an artifactual removal of several genes as horizontal gene transfer events or sequence contamination.

The new version of the manuscript includes an additional figure showing the effects of not having this threshold versus using the default parameters of GenEra (Fig. 3D), demonstrating that the implementation of this filter makes sense in the overall rationale of the pipeline.

We also rephrased this part of the manuscript to be more explicit on the rationale behind this parameter (lines 244-262):

“*The absence of genomic data in certain taxonomic levels can be erroneously interpreted as systematic gene loss events that can lead to inconsistent phylogenetic patterns that are artificially introduced by database limitations. Particularly, this applies for lineages with limited genomic data. For example, as of January 20th of 2023, the class Holothuroidea (sea cucumbers) has 15 sequenced genomes uploaded to the NCBI database, where only one (Apostichopus japonicus) was uploaded with gene annotations (46). Nonetheless, 5555 proteins in the NCBI belonging to Holothuroidea do not correspond to Apostichopus japonicus. These are mainly mitochondrial proteins (5213 sequences), but also include nuclear proteins (312 proteins) that span Holothuroidea and four other nested taxonomic groups leading to Apostichopus japonicus. Retaining those taxonomic levels severely impacts the gene age estimations of Apostichopus japonicus, showing artificial patterns of gene loss for most of the genes in these taxonomic groups (Fig. 3D). To address this issue, GenEra searches the entirety of sequence matches that were retrieved with DIAMOND and only retains the taxonomic levels for which at least one representative species matches more than 10% of the proteins in the query species for further analyses. This threshold was empirically established to exclude the organisms in the NR that are represented by only a few genes and not by genomic data (Fig. S2). Using this threshold improves the detection of taxonomically-inconsistent patterns of gene presence/absence by collapsing the taxonomic levels that would otherwise increase the proportion of artificial gene-loss events in the analysis (Fig. 3D).*”

Third, I may be missing it, but the results described in the text - the assignments of gene age for the different focal species in the plants, animals, and fungi - do not seem to be available anywhere. Though the manuscript says that they are in Supplemental Information, I cannot find them anywhere (I only have Supp Tables 1-6, none of which contain this information). I also do not see them in the GitHub Repository. These must be available as a condition of publication. If I have missed them or some technical error has occurred in transmitting the files, please clarify/correct.

RESPONSE 7: We want to sincerely apologize to both reviewers for this issue. We intended to upload all these data to the submission system as tab-delimited text files within a single tarball (tar.gz). Unfortunately, the submission portal decompressed the tarball every time we uploaded it, making a mess with hundreds of submitted files, so we ended up not uploading them on our original submission. This was a mistake on our part and we acknowledge that the availability of these files is required for the reproducibility of our results. We have now integrated all these data into excel files to avoid further issues with the submission portal (Supplemental data 1-3). There, you will find all the individual gene age assignments and the estimation of gene-family founder events, both before and after correcting for homology detection failure, for each of the 30 analyzed species. We also added a list of genes with ambiguous age estimations per species (genes with taxonomic representativeness < 30) and the summary of gene age estimations/founder events per species.

In an effort to be transparent with our results, we also uploaded another excel file (Supplemental data 4) where you will find the input tables that were used to generate the plots from Figure 4, and how we fitted species with different taxonomic levels into a single plot for each kingdom. This fitting process was made to convey our results in a visually comprehensive manner, and this process does not change our conclusions in any way, as we focused our discussion on the taxonomic levels that are shared between all or most of the analyzed species.

One more minor concern:

-	I am not clear on the biological rationale behind the TR score, the authors' method of filtering out cases of HGT or sequence contamination. It seems to me that the relevant quantity should be something like the number of independent losses that would have had to take place for the gene to have been present in the common ancestor of the distant homolog-possessing taxon and the query species: if this number is high, i.e. a large fraction of all the taxa under consideration would have to have lost the gene, such losses are implausible, and the data point to HGT/contamination. It is not totally clear to me from the description that the TR score amounts to this. It instead counts the percent of monophylys in which the gene is present/absent. While these are the same in the simple, ladder-like topology of Figure 2B, it is not obvious to me that they will always be identical in real phylogenies. I may be wrong; if so, clarification in the text would be useful. If I am correct,
can the authors clarify or say something about this choice?

RESPONSE 8: The reviewer is correct: our calculation of TR is based on the percentage of nested monophylies where the gene is present or absent. Albeit simple, this vision of a ladder-like topology should be valid for any real phylogeny as long as the taxonomic levels that are analyzed represent monophyletic groups (see RESPONSE 1). We generated a figure using the phylogeny of Li *et al.* (2021) to exemplify how this topology assumption is applicable even for complex tree topologies (Fig. S3; lines 935-942):

“*Figure S3. Example of a ladder-like topology when dealing with monophyletic groups. We use the phylogeny of Fungi published by Li et al. (94) to depict the way GenEra interprets evolutionary relationships between species, with S. cerevisiae as the query species. As long as the taxonomic ranks represent monophyletic groups, the evolutionary relationships between species can be interpreted as a ladder-like topology, where all the inner nodes within each taxonomic rank are treated as polytomies. Therefore, only the nodes that define the taxonomic groups become relevant for the assessment of gene ages and the calculation of taxonomic representativeness scores.*”

There are of course more sophisticated ways in which we could calculate a TR score. For example, our calculation does not account for the differential number of species that are contained within each taxonomic level (*e.g.*, in Figure S3, you would expect way more hits at the Saccharomyceta level compared to the *Saccharomyces* level), but just whether even a single hit is found or not at each level. The suggestion made by the reviewer would certainly account for more complex evolutionary scenarios of gene loss, and could even account for differential species diversity within each taxonomic level, making it more robust to complex scenarios. Nonetheless, its implementation would require us to either have an *a priori* estimation of the number of representative species for each taxonomic level, or for us to estimate the number of complete representative genomes that are available within each taxonomic level for any given database and then compare that to the total number of hits that were found for each gene at each taxonomic level. The reviewer will realize that this would require additional assumptions and a lot more computation time.

We argue that our TR score, despite being simple, is fast to calculate and it is efficient in finding inconsistent homology patterns, such as genes that are only found in a query eukaryotic genome and on bacteria or on organisms from other kingdoms, which are common types of contamination in genomic databases (Nevers *et al*., 2022). We incorporated all this information into the new version of the manuscript (lines 274-277 and 298-300):

“*This metric assumes a ladder-like phylogenetic topology between the query species and the database species at each taxonomic level, a condition that is always met as long as the taxonomic levels that classify the query species represent monophyletic groups (Fig. S3).*”

“*Low levels of taxonomic representativeness are expected for cases of cross-kingdom and cross-domain contamination that are pervasive in genomic databases (97).*”

**REFERENCES**

1. **James, J. E., Willis, S. M., Nelson, P. G., Weibel, C., Kosinski, L. J., & Masel, J. (2021). Universal and taxon-specific trends in protein sequences as a function of age. *Elife*, *10*, e57347.**
2. **Julian, A. T., Mascarenhas dos Santos, A. C., & Pombert, J. F. (2021). 3DFI: a pipeline to infer protein function using structural homology. *Bioinformatics Advances*, *1*(1), vbab030.**
3. **Li, Y., Steenwyk, J. L., Chang, Y., Wang, Y., James, T. Y., Stajich, J. E., *et al.* (2021). A genome-scale phylogeny of the kingdom Fungi. *Current Biology*, *31*(8), 1653-1665.**
4. **Madera, M., & Gough, J. (2002). A comparison of profile hidden Markov model procedures for remote homology detection. *Nucleic Acids Research*, *30*(19), 4321-4328.**
5. **Moody, L. A., Saidi, Y., Gibbs, D. J., Choudhary, A., Holloway, D., Vesty, E. F., ... & Coates, J. C. (2016). An ancient and conserved function for Armadillo‐related proteins in the control of spore and seed germination by abscisic acid. *New Phytologist*, *211*(3), 940-951.**
6. **Moyers, B. A., & Zhang, J. (2015). Phylostratigraphic bias creates spurious patterns of genome evolution. *Molecular biology and evolution*, *32*(1), 258-267.**
7. **Moyers, B. A., & Zhang, J. (2018). Toward reducing phylostratigraphic errors and biases. *Genome Biology and Evolution*, *10*(8), 2037-2048.**
8. **Nevers, Y., Rossier, V., Train, C., Altenhoff, A. M., Dessimoz, C., & Glover, N. (2022). Multifaceted quality assessment of gene repertoire annotation with OMArk. *bioRxiv*, 2022-11.**
9. **Pearson, W. R., Li, W., & Lopez, R. (2017). Query-seeded iterative sequence similarity searching improves selectivity 5–20-fold. *Nucleic Acids Research*, *45*(7), e46-e46.**
10. **Ryan, J. F., & Baxevanis, A. D. (2007). Hox, Wnt, and the evolution of the primary body axis: insights from the early-divergent phyla. *Biology Direct*, *2*(1), 1-11.**
11. **Sebé-Pedrós, A., Roger, A. J., Lang, F. B., King, N., & Ruiz-Trillo, I. (2010). Ancient origin of the integrin-mediated adhesion and signaling machinery. *Proceedings of the National Academy of Sciences*, *107*(22), 10142-10147.**
12. **Weisman, C. M., Murray, A. W., & Eddy, S. R. (2020). Many, but not all, lineage-specific genes can be explained by homology detection failure. *PLoS biology*, *18*(11), e3000862.**
13. **van Kempen, M., Kim, S., Tumescheit, C., Mirdita, M., Söding, J., & Steinegger, M. (2022). Foldseek: fast and accurate protein structure search. *bioRxiv*.**
14. **Varadi, M., Anyango, S., Deshpande, M., Nair, S., Natassia, C., Yordanova, G., *et al*. (2022). AlphaFold Protein Structure Database: massively expanding the structural coverage of protein-sequence space with high-accuracy models. *Nucleic acids research*, *50*(D1), D439-D444.**

**Second round of review**

**Reviewer 1**

The authors have addressed by concerns and I have no further comments. Hence I recommend accepting this manuscript.

**Reviewer 2**

The authors have responded to my suggestions to my satisfaction. Thank you for the clarification of the methodological issues in the text and for the inclusion of the supplementary information. I especially appreciate the incorporation of a profile-based method in the text and in the software package. All told, I think this will be of enormous utility to the field, and thank the authors for their work. I recommend publication at this stage.
